# A self-assembled trimeric protein vaccine induces protective immunity against Omicron variant

**DOI:** 10.1038/s41467-022-33209-9

**Published:** 2022-09-17

**Authors:** Cai He, Jingyun Yang, Weiqi Hong, Zimin Chen, Dandan Peng, Hong Lei, Aqu Alu, Xuemei He, Zhenfei Bi, Xiaohua Jiang, Guowen Jia, Yun Yang, Yanan Zhou, Wenhai Yu, Cong Tang, Qing Huang, Mengli Yang, Bai Li, Jingmei Li, Junbin Wang, Haiying Que, Li Chen, Wenyan Ren, Dandan Wan, Jiong Li, Wei Wang, Guobo Shen, Zhiwei Zhao, Li Yang, Jinliang Yang, Zhenling Wang, Zhaoming Su, Yuquan Wei, Xiaobo Cen, Yoshimasa Tanaka, Xiangrong Song, Shuaiyao Lu, Xiaozhong Peng, Guangwen Lu, Xiawei Wei

**Affiliations:** 1 Laboratory of Aging Research and Cancer Drug Target, State Key Laboratory of Biotherapy and Cancer Center, National Clinical Research Center for Geriatrics, West China Hospital, Sichuan University, No. 17, Block 3, Southern Renmin Road, Chengdu, Sichuan 610041 China; 2 National Kunming High-level Biosafety Primate Research Center, Institute of Medical Biology, Chinese Academy of Medical Sciences and Peking Union Medical College, Yunnan, China; 3 National Chengdu Center for Safety Evaluation of Drugs, State Key Laboratory of Biotherapy and Cancer Center, West China Hospital, Sichuan University, Collaborative Innovation Center for Biotherapy, Chengdu, China; 4 Center for Medical Innovation, Nagasaki University, 1-7-1 Sakamoto, Nagasaki, 852-8588 Japan; 5 State Key Laboratory of Medical Molecular Biology, Chinese Academy of Medical Sciences, School of Basic Medicine, Peking Union Medical College, Beijing, China

**Keywords:** SARS-CoV-2, Viral infection, Protein vaccines, Protein vaccines

## Abstract

The recently emerged Omicron (B.1.1.529) variant has rapidly surpassed Delta to become the predominant circulating SARS-CoV-2 variant, given the higher transmissibility rate and immune escape ability, resulting in breakthrough infections in vaccinated individuals. A new generation of SARS-CoV-2 vaccines targeting the Omicron variant are urgently needed. Here, we developed a subunit vaccine named RBD-HR/trimer by directly linking the sequence of RBD derived from the Delta variant (containing L452R and T478K) and HR1 and HR2 in SARS-CoV-2 S2 subunit in a tandem manner, which can self-assemble into a trimer. In multiple animal models, vaccination of RBD-HR/trimer formulated with MF59-like oil-in-water adjuvant elicited sustained humoral immune response with high levels of broad-spectrum neutralizing antibodies against Omicron variants, also inducing a strong T cell immune response in vivo. In addition, our RBD-HR/trimer vaccine showed a strong boosting effect against Omicron variants after two doses of mRNA vaccines, featuring its capacity to be used in a prime-boost regimen. In mice and non-human primates, RBD-HR/trimer vaccination could confer a complete protection against live virus challenge of Omicron and Delta variants. The results qualified RBD-HR/trimer vaccine as a promising next-generation vaccine candidate for prevention of SARS-CoV-2, which deserved further evaluation in clinical trials.

## Abstract (web summary)

The SARS-CoV-2 Omicron variant has quickly become the predominant circulating variant, due to the high transmissibility and immune escape. Here, the authors develop a trimeric protein vaccine candidate and show a sustained humoral immune response, and protection from challenge (Omicron and Delta) in various animal models.

## Introduction

The recently emerged Omicron (B.1.1.529) variant, which possess a large number of mutations, has rapidly outcompeted Delta (B.1.617.2) and become the predominant circulating SARS-CoV-2 variant^1^. Despite attenuated pathogenicity^2^, Omicron has higher transmissibility than Delta, with the number of Omicron infections dramatically increasing. The Omicron variant harbors the highest number of amino acid substitutions, deletions, and insertions in the spike glycoprotein, raising grave concerns that it may escape immune protection induced by currently marketed vaccines developed based on the original SARS-CoV-2 stains. As a matter of fact, Omicron variant was observed to show extensive resistance to many widely used COVID-19 vaccines with compromised neutralization antibodies^1,3–7^. More and more breakthrough infections caused by the Omicron variant increased in highly vaccinated populations^8^. Even the previously infected individuals are still exposed to the risk of re-infection^9^. Several studies reported that the serum neutralizing activities against Omicron could be reduced by 60- to 80-fold in convalescents, and by 20- to 130-fold in the vaccinated^10–14^. Some noticeable mutations, including K417N, G446S, E484A, S371L, N440K and Q493R in Omicron, are all shown to contribute to the reduction of antibody neutralization^1,4^. Therefore, vaccines designed to match the Omicron variant are constantly updated^14–16^. However, a recent study reported that Omicron RBD protein showed reduced antigenicity by inducing impaired serologic responses^17^. Moreover, an Omicron-mRNA booster shot did not provide any advantage in recalling high titers of neutralizing antibodies when compared to that using the currently available mRNA-1273 vaccine based on ancestral SARS-CoV-2^14^. These urgent issues prompted us to develop a new generation of SARS-CoV-2 vaccine that is able to confer robust protection against Omicron-included SARS-CoV-2 variants.

We and others have already shown that the spike RBD of SARS-CoV-2 represents a feasible immunogen for subunit vaccine development^18–21^. To further improve the antigenicity, multiple strategies for antigen multimerization targeting RBD, either by using a multimerization tag or by displaying on a proteinseneous nanoparticle, have been intensively investigated^21–25^, results from which indeed showed enhanced immunogenicity and improved protection. Nevertheless, these RBD multimerization trials almost inevitably introduced exogenous sequences in the design, which could dramatically complicate their potential clinical use. Previous studies have reported that peptides harboring the spike heptad-repeat sequences 1 and 2 (HR1 and HR2) could automatically assemble into a 6-helix bundle structure^26,27^, which reminds us that using this self-assembly feature of HR sequences should be able to trimerize RBD, potentially enhancing the antigenicity. In addition, the baculovirus expression system was chosen to express the various proteins used in our study. This technology was widely used in vaccine productions, including vaccines against human papilloma virus and influenza currently in use in Europe and the USA^28,29^. In the present study, we showed that HR-fused RBD protein that contains both L452R and T478K mutations could self-assemble into trimers, and elicit high levels of neutralizing antibodies to prevent infection of Omicron-included SARS-CoV-2 variants.

## Results

### Characterization of the recombinant RBD-HR/trimer protein

**Fig. 1:**
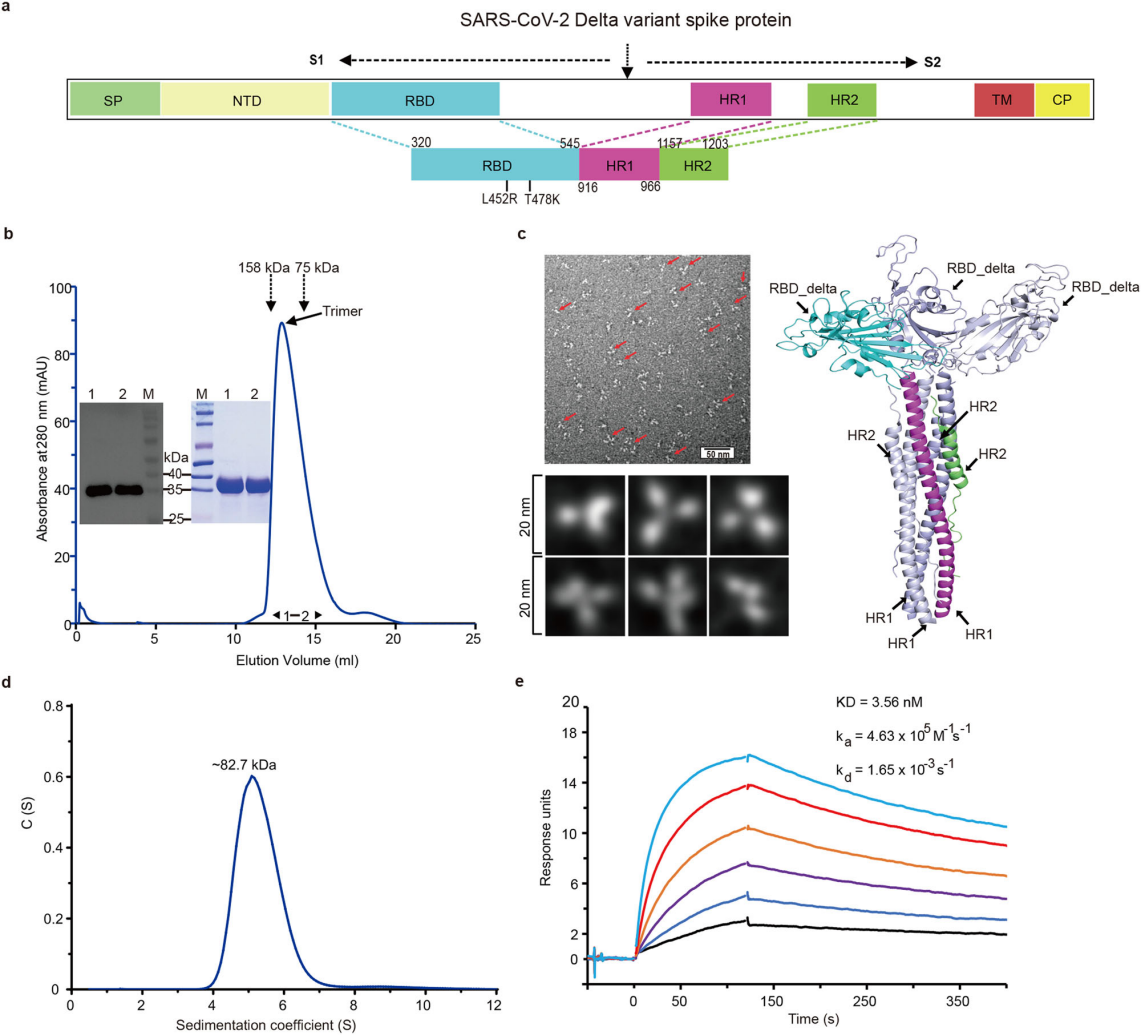
Characterization of the recombinant RBD-HR/trimer protein. **a** The schematic representation of the SARS-CoV-2 Delta variant spike protein. Our RBD-HR/trimer protein includes a RBD (320–545 aa) derived from the Delta variant (containing L452R and T478K mutations) and HR1 (916–966 aa) and HR2 (1157–1203 aa) domain in subunit S2 of spike protein. SP signal peptide, NTD N-terminal domain, RBD receptor binding domain. HR1 and HR2 heptad repeats 1 and 2, TM transmembrane domain, CP cytoplasmic domain. **b** A representative elution chromatograph of the recombinant RBD-HR/trimer protein using a calibrated Superdex Increase 200 column. The SDS-PAGE and Western blotting analyses of the eluted RBD-HR/trimer protein were shown. mAU milli-absorbance units, M marker; 1, the eluted sample of the ascending part of the protein peak; 2, the eluted sample of the descending part of the peak. **c** Transmission electron micrographs (left) and a molecular model (right) of the RBD-HR/trimer protein with a bouquet-like shape. RBD-Delta is displayed in blue, HR1 in purple, and HR2 in green. **d** The analytical ultracentrifugation (AUC) assay of the recombinant RBD-HR/trimer protein. **e** The real-time binding profile between purified RBD-HR/trimer protein and receptor ACE2 was performed by surface plasmon resonance (Biacore). Scale bar represents 50 nm in (**c**). Source data are provided as a Source Data file.

Our antigen design includes an RBD (320–545 aa) sequence derived from the Delta variant as well as an HR1 (916–966 aa) and an HR2 (1157–1203 aa) sequences derived from the SARS-CoV-2 S2 subunit, which are directly linked in a tandem manner (Fig. 1a). In light of the rigid-body structures for RBD and the HR six-helix bundle, we rationalized that a linker of sufficient length should be included between the RBD subunit and the HR trimerization tag to guarantee the proper formation of the protein trimer. Thus, the RBD antigen truncated at position 545 could give an approximate 18-amino-acid linker between the RBD (which ends at about 527) and the HR tag. Here, we referred to this recombinant protein containing double mutations (L452R and T478K) as RBD-HR/trimer. The recombinant protein was subsequently prepared via the Bac-to-Bac Baculovirus Expression System and purified to homogeneity. On a calibrated Superdex 200 Increase column, the protein was eluted around 12.9 ml, indicating the formation of trimers (Fig. 1b). The transmission electron microscopy (TEM) imaging revealed a trimeric structure of recombinant RBD/HR protein (Fig. 1c). Noted that our design would cluster three RBD molecules together on one side and stack the HR1/HR2 helices on the other, and such protein-assembly mode should therefore lead to a bouquet-shaped trimer (Fig. 1c). Using analytical ultracentrifugation (AUC), we found that our protein was of about 82.7 kDa in solution (Fig. 1d). Because the AUC calculations take the candidate protein as a globular molecule but our designed trimer is theoretically of bouquet-like shape, we believe the observed apparent molecule weight better fits in with a protein trimer. We also investigated the binding of our protein to human ACE2 (the cellular receptor for SARS-CoV-2) via surface plasmon resonance biacore. As expected, potent interactions with typical slow-association/slow-dissociation kinetics were recorded (Fig. 1e). The determined binding affinity was about 3.56 nM, coinciding well with our and others’ previous calculations^18,30^. The results demonstrated that trimerization of the RBD in our design would not interfere with the proper RBD-folding or with the solution exposure of the receptor binding motif. In the following text, we referred to this trimeric recombinant protein containing double mutations (L452R and T478K) as RBD-HR/trimer.

### Sustained humoral immune response and broad-spectrum neutralization elicited by the RBD-HR/trimer vaccine

**Fig. 2:**
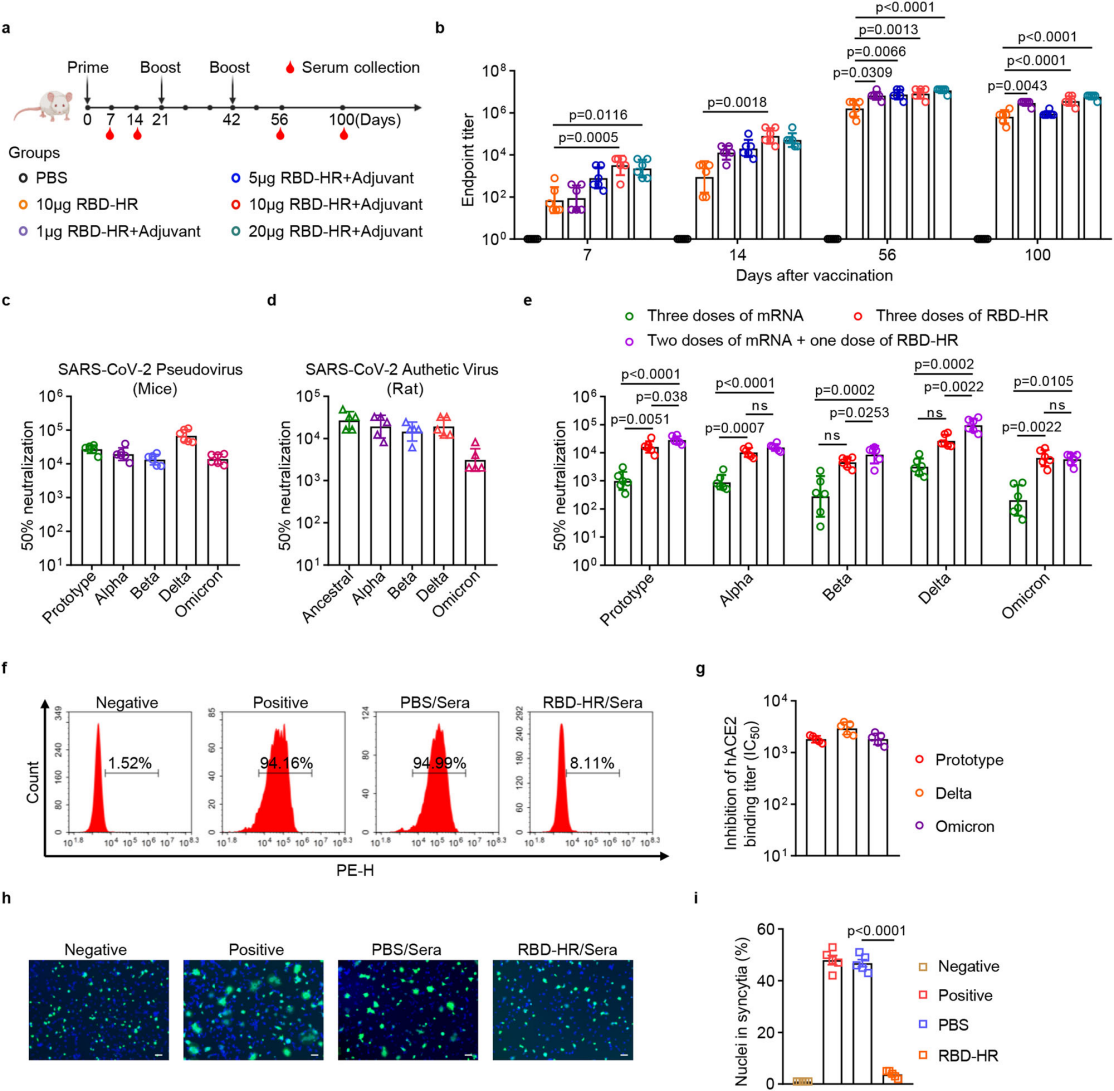
Induction of sustained humoral immune response with broad-spectrum neutralizing activities by RBD-HR/trimer vaccine. **a** The schematic representation of the mouse immunization and sample collection protocol. **b** The assay of endpoint titers of anti-RBD IgG induced by RBD-HR/timer vaccine was performed by ELISA (*n* = 6 mice per group). **c** Neutralizing antibody titers against pseudoviruses in sera from *n* = 6 mice vaccinated with 10 μg adjuvant-formulated RBD-HR/trimer vaccine were determined by pseudovirus neutralization assay. **d** Neutralizing antibody titers in sera from *n* = 5 rats immunized with 60 μg of RBD-HR/trimer vaccine against authentic SARS-CoV-2 virus were determined. **e** Neutralization antibody titers of sera from mice immunized with three doses of mRNA, three doses of RBD-HR/trimer, or two doses of mRNA followed one dose of RBD-HR/trimer (*n* = 6 mice per group). **f** Representative graphs of flow cytometry represent blockade of RBD-Omicron binding to cell-surface human ACE2 receptor by the immunized sera. Negative control: cells stained with PE-conjugated anti-human IgG Fc antibodies only; Positive control: in the absence of sera; PBS/Sera: in the presence of sera from mice treated with PBS; RBD-HR/Sera: in the presence of sera from mice immunized with RBD-HR/trimer vaccine. **g** The flow cytometry analysis of the inhibition of binding between RBD-Prototype, RBD-Delta or RBD-Omicron with cell-surface receptor ACE2 in the presence of immunized sera (*n* = 5 biological replicates). Representative images (**h**) and quantitative analysis (**i**) of syncytia in the cell-cell fusion mediated by SARS-CoV-2 S protein, in the presence or absence of immunized sera (*n* = 5 mice per group). Serum samples in (**c**), and (**f**) to (**i**) were collected on day 56 after the first immunization, and mice sera in (**e**) were collected on day 84. Scale bars represent 100 μm in (**h**). Data are presented as geometric mean values ± SD in **b**–**e** and **g**, and presented as mean with SEM in **i**. *P* values in **b** were determined by One-way ANOVA followed by Dunnett’s multiple comparisons test, and in **e** and **i** were conducted by One-way ANOVA analysis followed by Tukey’s multiple comparisons test. Source data are provided as a Source Data file.

To assess the immunogenicity of RBD-HR/trimer, the antibody response induced by the protein was firstly determined. NIH mice were intramuscularly vaccinated with 1, 5, 10 or 20 μg of RBD-HR/trimer formulated with MF59-like adjuvant following a prime-boost regimen spaced 21 days apart (one initial injection on day 0, and two boosters on day 21 and 42, respectively). As control, the mice were injected with PBS or 10 μg RBD-HR/trimer protein without adjuvant. Serum samples were collected on day 7, 14, 56 and 100 since the first vaccination injection (Fig. 2a). The mice immunized with a single prime dose of the RBD-HR/trimer vaccine induced elevated RBD-specific IgG responses on day 7 (Fig. 2b). In the early immune stage, the binding antibody response showed dose-dependency such that higher anti-RBD IgG titers were observed in mice immunized with 10 μg and 20 μg adjuvant-formulated RBD-HR/trimer on day 7 and day 14 (Fig. 2b). Nevertheless, a low dose of 1 μg RBD-HR/trimer with adjuvant could also produce high levels of anti-RBD IgG after two doses of vaccinations. Even in the adjuvant-free group that only received RBD-HR/trimer vaccination, strong IgG antibody responses were observed after the final immunization (Fig. 2b). Noting that comparable endpoint titers of anti-RBD antibodies could be elicited with a range of vaccine doses after three shots, we chose, unless otherwise specified, 10 μg RBD-HR/trimer formulated with adjuvant for subsequent experiments.

In the next stage, pseudovirus neutralization assay was conducted with the sera from immunized NIH mice. As expected, the RBD-HR/trimer vaccine indeed elicited high titers of neutralizing antibodies (nAbs) in mice that could block the infection of all pseudoviruses (Fig. 2c). The geometric mean titers (GMTs) of 50% neutralization to prototype, Alpha, Beta, Delta and Omicron pseudoviruses were 27,227, 19,292, 13,312, 67,680 and 13,834, respectively. Furthermore, authentic virus neutralization assays were performed with the sera from immunized human ACE2 transgenic mice, rats, and non-human primates. Take the vaccinated rats for instance, the RBD-HR/trimer vaccine showed a good immunogenicity (Supplementary Fig. 1a, b) and elicited high neutralizing antibodies against a variety of variants in rats (Fig. 2d). The 50% neutralization GMTs to the Omicron variant was determined to be 3104, highlighting strong neutralization potency for authentic virus (Fig. 2d). Moreover, the neutralization capacities against authentic SARS-CoV-2 viruses in sera from vaccinated mice and non-human primates were described and presented in the subsequent section of “Complete protection against Omicron and Delta challenge by RBD-HR/trimer vaccination” (Fig. 5a, f).

We next investigated the effects of the RBD-HR/trimer vaccine as a heterologous booster dose in sequential immunization. We selected the messenger RNA (mRNA)-based vaccines which have been widely used globally and proven to be an effective platform for the prevention of SARS-CoV-2 infection^31,32^. Despite the effectiveness, the antibodies induced by the mRNA vaccine following the standard two-dose vaccination strategy were severely compromised when neutralizing SARS-CoV-2 variants^5^. It has been suggested that this challenge might be solved with a heterologous third-dose booster to enhance the immune response. To learn if our RBD-HR/trimer vaccine can be used as a candidate for heterologous third-dose booster, we designed a mRNA vaccine based on the sequence of SARS-CoV-2 Delta full-length spike protein. We then immunized mice with two injections of mRNA vaccine on day 0 and 21, followed by a third-dose immunization of the RBD-HR/trimer vaccine on day 42 (Supplementary Fig. 2a). In terms of the virus neutralization, the heterologous immunization regimen showed much improved neutralizing capacities against all variants in the panel than homologous mRNA vaccination. The GMTs of 50% neutralization in the homologous mRNA group against prototype, Alpha, Beta, Delta, and Omicron pseudoviruses were determined to be 993, 893, 281, 3182, and 204, respectively (Fig. 2e). For the heterologous vaccination group, the GMTs were observed to increase by 28.6-, 17.4-, 30.5-, 30.2-, and 28.8-fold, respectively. Taken together, these results demonstrated that the recombinant RBD-HR/trimer antigen could be developed as a protein-subunit vaccine or be used as an extra booster shot to prevent the infection of Omicron-included SARS-CoV-2 variants with broad-spectrum protection.

In order to learn the underlying mechanism of virus neutralization, the binding of RBD to cell-surface ACE2 and the cell-cell fusion mediated by spike were tested with the vaccinated sera, In the flow cytometric analyses, about 94.16% of the ACE2-expressing HEK-293T cells could be stained with RBD-Omicron in the absence of the immune serum. As expected, no blocking of staining was observed with the serum from mice immunized with PBS (Fig. 2f). In contrast, the binding between RBD-Omicron and ACE2 could be almost completely blocked after incubation with the immune serum from RBD-HR/trimer vaccinated mice at up to 1:270 dilution (Fig. 2f). Similar ACE2-binding inhibition could also be recorded for RBD-Delta and RBD-Prototype, though the inhibition titers for RBD-Delta were slightly higher (Fig. 2g). In the cell-cell fusion assay, the syncytium-formation mediated by spike could be completely inhibited by the immune serum from RBD-HR/trimer vaccinated mice. The serum of the PBS group, however, showed no blocking effect (Fig. 2h, i). These results indicated that the serum antibodies elicited by the vaccine neutralized virus infection by blocking receptor binding and inhibiting subsequent virus-cell membrane fusion.

### Mutations of L452R and T478K both benefit vaccination-induced antibody response against Omicron and Delta variants

**Fig. 3:**
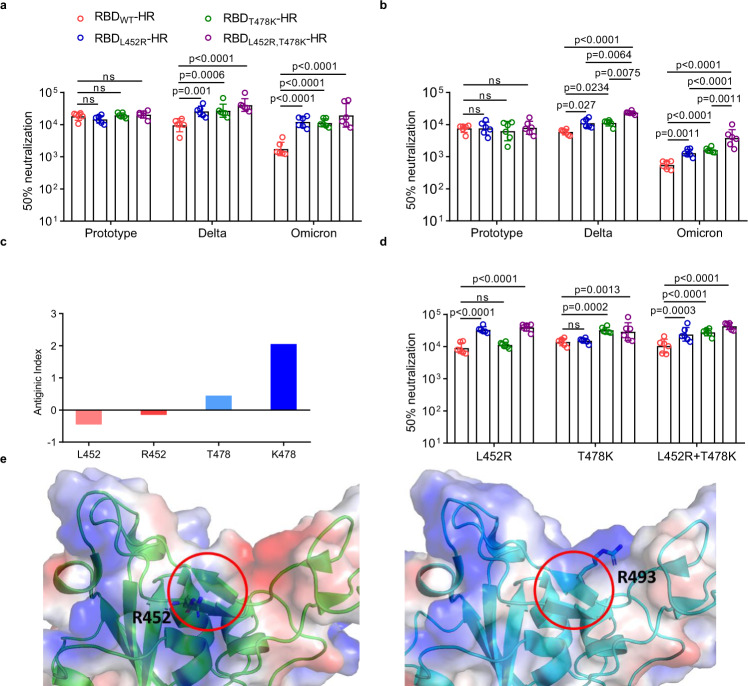
Mutations of L452R and T478K both benefit to antibody response against Omicron and Delta variants. **a** NIH mice were immunized with 10 μg of adjuvant-formulated RBD_WT_-HR, RBD_L452R_-HR, RBD_T478K_-HR, and our RBD-HR/trimer, serum samples were collected on day 56 to assay the neutralizing antibodies against prototype, Delta and Omicron pseudovirus (*n* = 6 mice per group). **b** Neutralizing antibody titers in vaccinated mice after two doses of immunization (*n* = 6 mice per group). **c** Antigenicity analyses of the SARS-CoV-2 original-strain and Delta-variant RBDs using the Jameson-Wolf method. The antigenic index values for the L452/R452 and T478/K478 pairs are recorded and further presented using histograms. **d** Neutralizing antibody titers induced by RBD_WT_-HR, RBD_L452R_-HR, RBD_T478K_-HR, and RBD-HR/trimer against mutated psudoviruses containing signal mutation of L452R, T478K or double L452R + T478K substitutions (*n* = 6 mice per group). **e** The spike RBD structures highlighting the small two-stranded β-sheet in the middle of the external subdomain. The structures of the Delta (PDB code: 7wbq) and Omicron (PDB code: 7wbp) RBDs are presented in the same orientation and further shown in the left and right panels, respectively. The Delta-specific residue of R452 and omicron-specific residue of R493 are marked. The two anti-parallel β-strands where R452 and R493 locate are highlighted by encircling with red lines. Data are presented as geometric mean values ± SD in **a**, **b** and **d**. *P* values were conducted by Two-way ANOVA analysis followed by Tukey’s multiple comparisons test. Source data are provided as a Source Data file.

It is inspiring that our RBD-HR/trimer vaccine, which contains the L452R and T478K substitutions simultaneously, is very effective against Omicron such that the vaccine immunization of multiple animals can induce high neutralizing antibody titers against Omicron variant (Fig. 2c–g). To learn the impact of the individual mutations of L452R and T478K on the vaccination-induced antibody response against Omicron, we further prepared the HR-fused wild-type RBD protein (RBD_WT_-HR) and the RBD-HR proteins with the L452R (RBD_L452R_-HR) and T478K (RBD_T478K_-HR) substitutions, respectively (Supplementary Fig. 3a–c). Mice were then immunized with the adjuvant-formulated proteins in parallel with three injections and sera were subsequently collected for comparison of neutralizing capacity using the pseudoviruses. For RBD_WT_-HR, the GMTs of 50% neutralization for both Delta and Omicron variants, especially for Omicron variants, were apparently reduced, compared to that for the prototype virus. However, the serum antibodies of the RBD_T478K_-HR-vaccinated group exhibited improved neutralization against both Omicron and Delta variants, showing an increase in GMT by 6.43- and 2.82-fold, respectively, compared with vaccination with RBD_WT-_HR protein (Fig. 3a). Such increase coincides well with the fact that both the Omicron and Delta variants contain the T478K mutation in spike. Unexpectedly, immunization with RBD_L452R_-HR not only enhanced the neutralization capacity against Delta (GMT increased by 2.70-fold), but also improved neutralization for Omicron (GMT increased by 6.86-fold) (Fig. 3a). Remarkably, the immunization with our RBD-HR/trimer with both L452R and T478K mutations, could have apparently elevated levels of the neutralizing antibodies for Omicron and Delta, compared with the vaccine with single mutation after two doses, at early stage of the immunization (Fig. 3b), highlighting a synergistic effect between the two substitutions towards improved efficacy against Omicron and Delta in a quicker way.

To learn the potential impact of the L452R and T478K mutations on the RBD antigenicity, we utilized the Jameson and Wolf algorithm^33,34^ to calculate their antigenic indices, which could generate surface contour profiles and quantitatively predict protein antigenicity from the amino acid sequence. Bioinformatic analyses of the RBD sequences revealed that both L452R and T478K mutations seem to benefit RBD antigenicity, raising the antigenic index values from −0.45 for L452 to −0.15 for R452 and from 0.45 for T478 to 2.05 for K478, respectively (Fig. 3c). Furthermore, we used the pseudovirus with L452R or T478K mutation alone or both mutations to confirm whether the improved neutralization against Omicron and Delta variants may result from the mutations-specific or non-specific to our vaccine. RBD_T478K_-HR vaccine selectively induced the enhanced neutralizing antibody against the pseudovirus with T478K or double L452R + T478K substitutions, compared with the prototype pseudovirus or those with L452R substitutions (Fig. 3d). Also, RBD_L452R_-HR vaccine selectively induced the enhanced neutralizing antibody against the pseudovirus with L452R or double L452R + T478K substitutions, compared with the prototype pseudovirus or those with T478K substitutions (Fig. 3d). Although there is no L452R mutation on the Omicron variant, we noticed that both the Delta-specific L452R mutation and the Omicron-specific Q493R mutation were located on a small two-stranded β-sheet in the middle of the RBD external subdomain (Fig. 3e). In addition, both mutations introduced a basic guanidine group to the β-sheet. The two amino acid substitutions might result in certain similarities in the epitope characteristics targeting the β-sheet, benefiting cross neutralization of RBD_L452R_-HR immunized sera against Omicron.

### Strong T cellular immune response induced by RBD-HR/trimer in vivo

**Fig. 4:**
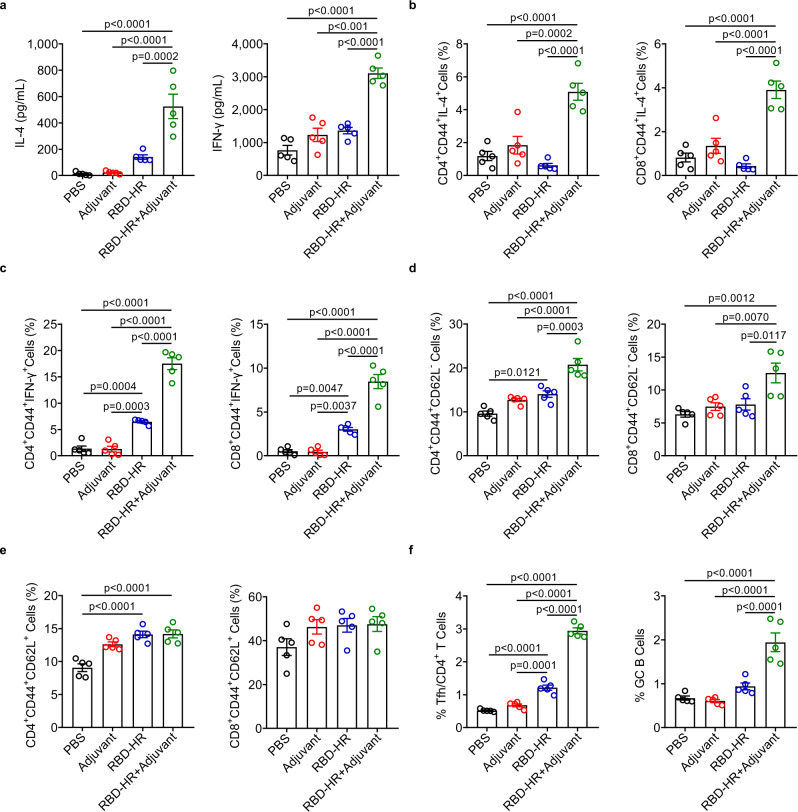
RBD-HR/trimer vaccine induced strong T cell immune response in vivo. **a** The levels of IFN-γ and IL-4 in the supernatants of spleen lymphocytes from immunized mice were detected by ELISA after stimulation with RBD (10 μg/ml) for three days. **b**, **c** The number of RBD-specific IL-4 or INF-γ-producing memory T cells in spleen were analyzed by flow cytometry after stimulation with RBD. Memory T cells were gated on CD44^high^ B220^−^MHC-II^−^ CD4^+^ or CD8^+^. **d** The percentage of effector memory (CD44^+^CD62L^−^) of CD4 or CD8 T cells in spleen. **e** The percentage of central memory (CD44^+^CD62L^+^) of CD4 or CD8 T cells in spleen. **f** The frequency of T follicular helper cells (CD4^+^CXCR5^+^PD-1^+^) and germinal center B cells (CD19^+^GL7^+^CD95^+^) in spleen were analyzed by flow cytometry. *n* = 5 mice each group in **a**–**f**. Spleen samples in **a–f** were collected on day 49 after the first vaccination, and isolation of lymphocytes was performed as described in Methods. Data are presented as mean values ± SEM. *P* values were conducted by one-way ANOVA analysis followed by Tukey’s multiple comparison post hoc test in **a**–**f**. Source data are provided as a Source Data file.

Cellular immune responses play a critical role in the protection against pathogen infection in which both CD4+ and CD8+ T cells are involved^18^. Sufficient CD4^+^ T helper cell responses are required to elicit high-level antibody responses. Both CD4^+^ and CD8^+^ T-cell responses against spike protein of SARS-CoV-2 have been detected in COVID-19 patients, and this response is closely related to production of neutralizing antibodies in vivo^35,36^. Thus, we further investigated the role of our RBD-HR/trimer vaccine in activating T cell responses. The lymphocytes from spleen were isolated and then analyzed by ELISA and intracellular cytokine staining (ICS). The levels of IL-4 and INF-γ in cell supernatant were significantly increased after stimulation with RBD protein in the RBD-HR/trimer formulated adjuvant group (Fig. 4a). In addition, robust T cell responses induced by our vaccine were also manifested by increased numbers of RBD-specific IL-4 and INF-γ-producing memory T cells (including CD4^+^CD44^+^IL-4^+^, CD8^+^CD44^+^IL-4^+^, CD4^+^CD44^+^INF-γ^+^, and CD8^+^CD44^+^INF-γ^+^ cells) (Fig. 4b, c).

Memory T cells play an important role in the immune process, especially against pathogen re-infection. We then evaluated the proportion of central memory and effector memory T cells in spleen on day 49 since the first vaccination. Subsets of CD4^+^ and CD8^+^ effector memory T cells (CD4^+^CD44^+^CD62L^−^, CD8^+^CD44^+^CD62L^−^) were significantly elevated in mice vaccinated with adjuvant-formulated RBD-HR/trimer vaccine (Fig. 4d). However, the percentages of central memory T cells of both CD4^+^ and CD8^+^ subsets were not significantly increased after vaccination (Fig. 4e). These results indicated that our vaccine could enhance the cytotoxic CD8^+^ T lymphocytes and the CD4 T^+^ helper arm to protect against SARS-CoV-2 infection. The germinal center (GC) response is critical for the generation of affinity-matured plasma cells as well as the memory B cells that are capable of mediating long-term protective immunity^37^. In addition, a strong and persistent T follicular helper (Tfh) cell response elicited by vaccines could enhance the long-term immunity to prevent SARS-CoV-2 infection^38^. Thus, we next examined the effects of our RBD-HR/trimer vaccine on the formation of GC in spleen. As expected, immunization with RBD-HR/trimer with adjuvant remarkably increased the frequency of Tfh cells (CD4^+^CXCR5^+^PD-1^+^) and GC B cells (CD19^+^GL7^+^CD95^+^) in spleen (Fig. 4f). Taken together, these results demonstrated that high-magnitude T-cell responses can be induced by our RBD-HR/trimer vaccine to facilitate the production of high levels of neutralizing antibodies.

### Complete protection against Omicron and Delta challenge by RBD-HR/trimer vaccination

**Fig. 5:**
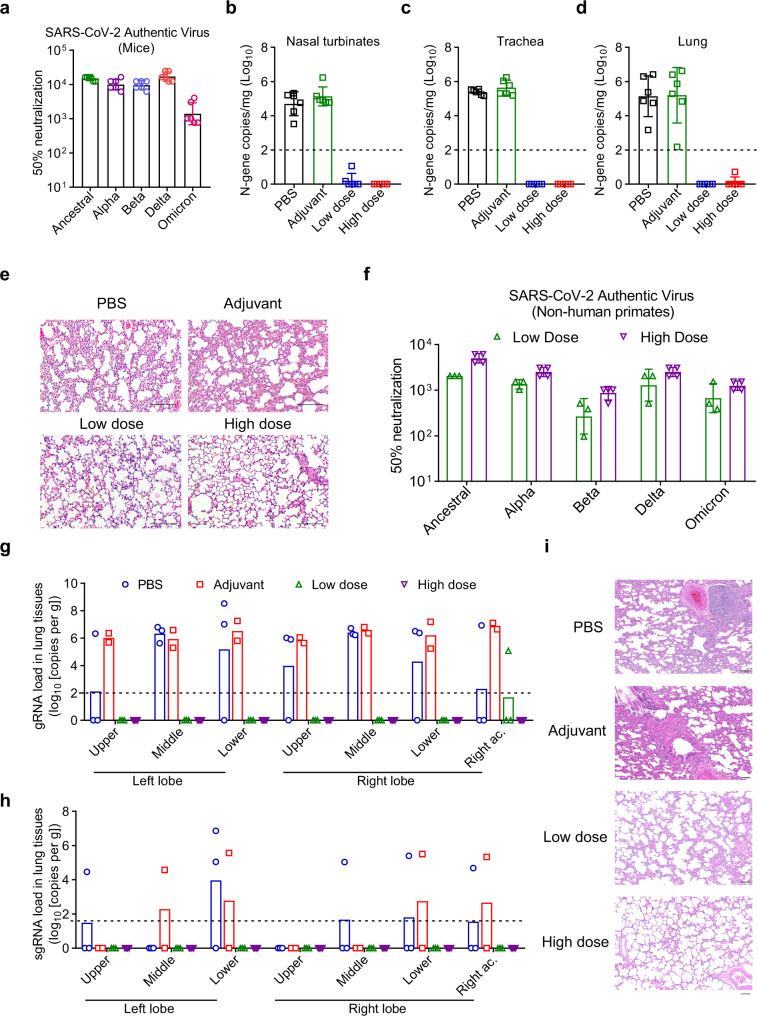
RBD-HR/trimer vaccine completely prevented the infection of Omicron and Delta variants. **a** Transgenic hACE2 mice with ICR background were immunized with 10 μg (low dose) or 20 μg (high dose) of adjuvant-formulated RBD-HR/trimer vaccine, the sera were collected and the neutralizing antibody titers against live SARS-CoV-2 (including ancestral, Alpha, Beta, Delta, and Omicron) in sera from vaccinated mice were determined by an authentic SARS-CoV-2 virus neutralization assay (n = 6 mice vaccinated with 10 μg RBD-HR/trimer vaccine). **b–d** Transgenic hACE2 mice immunized with RBD-HR/trimer were challenged with 1 × 10^5^ PFU of SARS-CoV-2 Omicron viruses. The nasal turbinates (**b**), trachea (**c**) and lung tissue (**d**) were collected on day 4 post infection to determine the levels of gRNA by RT-qPCR, *n* = 6 mice per group in **b**–**d**. **e** The histopathological changes in the lung tissue of hACE2 mice challenged with Omicron. **f** Non-human primates were immunized with 30 μg (low dose) or 60 μg (high dose) of RBD-HR/trimer vaccine. Non-human primates treated with PBS or adjuvant were used as control. The neutralizing antibody titers of sera from immunized non-human primates against authentic SARS-CoV-2 viruses were determined. Low-dose group: *n* = 3 non-human primates, High-dose group: *n* = 4 non-human primates. **g**, **h** The levels of gRNA (**g**) and sgRNA (**h**) in lung tissue of non-human primates on day 7 post infection were determined by RT-qPCR. **i** The pathological changes in the lungs of non-human primates were observed by hematoxylin and eosin staining. Scale bars represent 100 μm in (**e**) and (**i**). Data are presented as geometric mean values ± SD in **a**–**d** and **f**, and data are individual values and geometric means in **g** and **h**. Source data are provided as a Source Data file.

Next, we evaluated the in vivo efficacy of our RBD-HR/timer vaccine against Omicron, transgenic hACE2 mice were immunized with low dose (10 μg) or high dose (20 μg) of RBD-HR/trimer. Prior to the viral challenge, we collected the sera and evaluated the neutralizing capacity against authentic ancestral SARS-CoV-2 viruses. The results showed the 50% GMTs were 14,886, 10,033, 9563, 17,040, and 1408 for ancestral, Alpha, Beta, Delta, and Omicron viruses, respectively, indicating a high level of neutralization capacity (Fig. 5a).

The immunized mice were then challenged with 1 × 10^5^ plaque-forming units (PFU) of SARS-CoV-2 Omicron via intranasal routes. Multiple tissues of the respiratory system, including the nasal turbinates, trachea and lung tissues, were collected on day 4 post infection to assay viral loads. In nasal turbinates, PBS and adjuvant treated mice had geometric mean copy numbers of 5.03 × 10^4^ and 1.37 × 10^5^ copies of N gene/mg tissue, respectively (Fig. 5b). In trachea, the geometric mean copy numbers in the PBS and adjuvant groups were 2.48 × 10^5^ and 4.40 × 10^5^ copies of N gene/mg tissue (Fig. 5c). Although it was proposed that the tropism of Omicron might be shifted to the upper respiratory tract^39^, we still observed high levels of virus replication in lung tissue of unvaccinated animals (PBS: 1.40 × 10^5^ N copies/mg, adjuvant: 1.59 × 10^5^ N copies/mg tissue) (Fig. 5d). In contrast, the viral loads in nasal turbinates, tracheal, and lung tissues were undetectable in all mice vaccinated with adjuvanted RBD-HR/trimer (Fig. 5b–d). Although the pathogenesis of Omicron is less severe than those of other VOCs^2^, mild pathologic changes could still be observed, featuring multifocal areas of consolidation and mild alveolar septa thickening and alveolar congestion. In addition, small patches of inflammation composed of macrophage, neutrophiles and lymphocytes were occasionally observed adjacent to small blood vessels (Fig. 5e). By contrast, the lungs of all the vaccinated animals were histologically normal, displaying intact pulmonary alveoli structure, with no apparent inflammation (Fig. 5e).

We next evaluated the immunogenicity and protective capacity of RBD-HR/trimer in non-human primates (Supplementary Fig. 4a). High levels of binding and neutralizing antibodies against authentic SARS-CoV-2 variants were induced in non-human primates (Fig. 5f and Supplementary Fig. 4b). The GMTs for Omicron virus in low dose (30 μg) and high dose (60 μg) group were 671 and 1254, respectively, and the neutralizing antibodies induced by RBD-HR/trimer remains very effective against other SARS-CoV-2 authentic viruses, including ancestral, Alpha, Beta and Delta viruses (Fig. 5f). We performed the challenging infection of the live Delta variant virus in non-human primates, before Omicron variant virus became prevalent worldwide. While high levels of viral gRNA were detected in the control non-human primates, the viral gRNA was almost completely undetectable in the lung tissues of the vaccinated non-human primates (though the right accessory lobe of one non-human primate in the low-dose group showed detectable but significantly lower level of gRNA) (Fig. 5g). We also tested the sgRNA levels in the lung tissues, which features the active replication of the viruses. As expected, sgRNAs were detected only in the control non-human primates rather than in any of the vaccinated animals (Fig. 5h). In addition, histological examination showed that the lung tissues collected from control animals developed viral interstitial pneumonia. By contrast, the lungs of all the vaccinated animals showed no significant histopathological change without apparent inflammation (Fig. 5i). These data demonstrated that our RBD-HR/trimer protein is an effective candidate vaccine and can completely prevent the infection of Omicron-included SARS-CoV-2 variants in vivo, which deserves to be further evaluated in clinical trials.

## Discussion

The emergence of SARS-CoV-2 variants has been impairing population protection with the currently marketed vaccines. Notably, the recently emerged Omicron variant, which has numerous mutations that potentially enhance viral transmission and compromise neutralization, has been rapidly outcompeting Delta globally^40^. Vaccines based on Omicron variant also have the reduced ability to induce the neutralizing antibody responses^17^. In face of such an urgent situation, a new generation of SARS-CoV-2 vaccines that are effective against currently known VOCs, especially the Omicron variant, should be developed. In the present study, we reported the development of a self-assembled trimeric RBD subunit vaccine that can efficiently prevent the infection of Omicron-included SARS-CoV-2 variants. The vaccine antigen is designed by direct fusion of RBD with HR and the resultant RBD-HR protein can assemble into trimers in solution. The RBD-HR/trimer protein formulated with an adjuvant can elicit both sustained humoral and robust T-cell immune responses. Immunization with the RBD-HR/trimer vaccine induced high levels of neutralizing antibodies against the prototype, Alpha, Beta, Delta, and Omicron viruses and conferred complete protection against live virus challenge, including the Omicron and Delta variants in multiple animal models.

Previous studies have shown that RBD is able to induce high neutralizing antibodies as a protein vaccine with minimal side effects, and it could be manufactured at a low cost^18,19,21^. Several studies have proved that modified spike or RBD proteins, such as S trimer or RBD dimer, could generate more neutralizing antibodies, indicating that the polymeric protein vaccines might induce stronger humoral and cellular immune responses than monomeric antigens^19,22,24,41^. Multiple studies have reported the successful enhancement of RBD immunogenicity by using a multimerization tag or by cross-linking RBD monomer to a nanoparticle^21–25^. Nevertheless, these designs of RBD multimerization almost inevitably introduced non-SARS-CoV-2 amino acid sequences. Moreover, the protein production process for these antigens could be rather complex, requiring separate expression and purification expression, dramatically complicating their potential clinical use. Inspired by the fact that SARS-CoV-2 spike HR1 and HR2 could automatically assemble into a six-helix cluster structure via the strong hydrophobic interface among the HR units^26,27,42^, we have, by utilizing the self-assembly feature of HR, successfully improved the immunogenicity of RBD through trimerization without introducing any non-SARS-CoV-2 viral sequences.

Regarding the SARS-CoV-2 mRNA vaccines (mRNA-1273 and BNT162B2) that have been approved for emergency use around the world^31,32^, a significant reduction in neutralizing antibody titers against Omicron variants has been observed after the second dose vaccination of theses vaccines^5,6^. Although a third booster shot can improve the neutralizing activities, neutralization for the Omicron variant is still dramatically compromised^43^. Similar results were also observed in our homologous vaccination experiments with three consecutive injections of mRNA vaccines. Nevertheless, we notice that the RBD-HR/trimer vaccine, when used as an extra booster dose, could elicit much higher titers of neutralizing antibodies against Omicron-included variants after two injections of mRNA vaccines. Therefore, we believe that our RBD-HR/trimer vaccine represents a favorable candidate for urgent use as a heterologous third-dose booster to combat the Omicron variant.

We have previously reported that vaccines based on mutated proteins could better neutralize the corresponding mutant strain^44^. A recent study also demonstrated that the neutralizing antibody response could be the strongest against the variants which share the same mutations^45^. In the current study, we prove that the mutation at the T478K shared by both Delta and Omicron is critical for preserving protection effectiveness against the Omicron variant. Unexpectedly, mutation of L452R is also beneficial to the improved antigenicity. Several studies report that the neutralizing activities against Omicron could be reduced by 60- to 80-fold in convalescents, and by 20- to 130-fold in vaccinated individuals^10–14^. However, in the present study, compared with the prototype pseudovirus, there was only a slight decrease in neutralizing antibodies against Omicron variant in sera from vaccinated mice. In addition, the neutralizing antibody titers against the authentic Omicron viruses in sera from mice and rats reduced by 11.3- and 8.7-fold than the ancestral viruses, respectively. In non-human primates, compared with the authentic ancestral viruses, the neutralizing antibodies for Omicron variant only reduced by 3.05- and 4.00-fold in the low dose and high dose groups, respectively. Thus, in the present study, GMTs of 50% neutralization to Omicron variant maintained at very high levels: 13834 in mice in the Omicron pseudovirus neutralization test, and 1408 in mice, 3104 in rats, 1254 (high dose) and 671 (low dose) in non-human primates in the authentic Omicron variant virus neutralization test, respectively. A previous study has demonstrated that the levels of neutralizing antibodies in the vaccine recipients were directly correlated with the degree of vaccine efficacy, and 50% pseudovirus neutralization titer value of 10, 100, and 1000 corresponded to estimated vaccine efficacies of 78%, 91% and 96%, respectively^46^. Therefore, we speculated that robust protection against Omicron-included variants could be induced by our RBD-HR/trimer vaccine in clinical trials.

The Omicron subunit proteins may not be an appropriate selection for developing subunit vaccines against Omicron-included SARS-CoV-2 variants. We and others have shown that recombinant S1 and RBD proteins derived from Omicron only elicited impaired serologic responses with limited neutralizing activities^17,47^. Omicron RBD itself also shows reduced thermostability^48^. In addition, a recent study revealed that an Omicron-mRNA booster shot seems to not provide any advantages in recalling high titers of neutralizing antibodies when compared to that using the currently available mRNA-1273 vaccine^14^. It is especially worth noting that infection with Delta or immunization with Delta-matched vaccine elicited an effective cross-variant neutralization, whereas Omicron infection or immunization with Omicron-matched vaccine only induced a limited cross-neutralizing humoral immune response^49^. Echoing these observations, our results clearly show that the RBD-HR protein derived from Delta variant outperforms the WT protein in the induction of cross-Omicron immune response and that the Delta-specific L452R and T478K mutations both benefit the cross neutralization. It is also noteworthy that the HR domain might also potentially induce neutralizing immune response^50^. Considering that HR is highly conserved among SARS-CoV-2 variants, we believe the RBD-HR/trimer antigen represents a good subunit vaccine candidate for broad-spectrum protection of SARS-CoV-2 infection. Therefore, our vaccine design has provided a new strategy to increase the immunogenicity of RBD without introducing non-viral components. The RBD-HR/trimer vaccine could be used either as a stand-alone vaccine or as an extra booster shot in humans to protect against the circulating Omicron-included SARS-CoV-2 variants.

## Methods

### Cell culture

HEK-293T cells (CRL-11268) were purchased from the American Type Culture Collection (ATCC). 293T cells stably expressing ACE2 (293T/ACE2) were generated in our laboratory as previously reported^18^. We maintained the cells in complete Dulbecco’s modified Eagle’s medium (DMEM, Gibco, USA), which was supplemented with 10% fetal bovine serum (FBS, PAN-Biotech, Germany), 100 μg/ml streptomycin, and 100 U penicillin (Gibco, USA) at 37 °C with 5% CO_2_. Sf9 cells (ATCC CRL-1711) were maintained in SIM SF medium (Sino Biological) at a non-humidified shaker at 28 °C.

### Gene cloning, expression and protein purification

The RBD-HR/trimer protein used in this study was expressed using the Bac-to-Bac baculovirus expression system (Invitrogen). Because the C538 residue in RBD might form inter-chain disulfide bond and subsequently lead to dimerization of the RBD subunit, a combination of the inter-chain disulfide between the RBD subunits and the trimerization HR tag fused at the C-terminus of RBD could cause irregular protein aggregation. Therefore, the C538 residue was substituted with a serine residue (C538S) to guarantee the proper formation of the protein trimer. Subsequently, our construct design features with the direct linkage of the S-RBD sequence of the SARS-CoV-2 Delta strain (amino acids V320-G545 with L452R and T478K, as well as C538S) to an HR1 (amino acids L916-L966) and an HR2 sequence (amino acids K1157-L1203) of the SARS-CoV-2 S2 subunit. This designed protein sequence is further fused at its N-terminal and in a tandem manner with the sequences of a GP67 signal peptide to guarantee protein secretion, a Trx tag to help protein folding, a 6xHis tag to facilitate protein purification, and an enterokinase (EK) cleavage site for tag removal^51^.

For gene cloning, the coding sequence described above was first amplified and then incorporated into the pFastBac1 vector via the BamHI and HindIII restriction sites. The sequencing-verified plasmid was subsequently transformed into *Escherichia coli* DH10b cells to generate recombinant bacmids.

For protein expression, the bacmid was first transfected into Sf9 insect cells using LipoInsect Transfection Regent (Beyotime Biotechnology, Shanghai, China) according to the manufacturer’s instructions. The cell culture supernatants, which contained the packaged recombinant baculoviruses, were harvested about 72 h post transfection. The baculovirus was then passaged in Sf9 cells for 2–3 times. The amplified baculovirus was subsequently inoculated into the Sf9 cells for protein production.

For protein purification, the culture supernatants from the Sf9 cells were collected about 72 h after infection and passed through a 5 ml HisTrap excel column (GE Healthcare) for primary purification. The recovered proteins were further cleaved using the EK protease at a ratio of 1U:1 mg protein at 4 °C for about 18 h. The cleaved tag was then removed by affinity chromatography using the HisTrap excel column (GE Healthcare). The flow-through solution, which contains the RBD-HR/trimer protein, was subsequently collected, concentrated, and loaded onto a Superdex 200 Increase 10/300 GL column (GE Healthcare) for further purification. The purity of the protein was determined by SDS-PAGE and visualized by staining with Coomassie blue and by western blotting using the SARS-CoV-2(2019-nCoV) spike antibody (Sino Biological, 40591-MM42, 1:2000). The western blot data were collected using Clinx ChemiScope Series with ChemiCapturePAD V2018.5 (Clinx Science Instrument Co., Ltd).

### Negative-staining transmission electron microscopy (TEM) imaging and processing

For sample preparation, 3 μl of RBD-HR/trimer protein was transferred to the shiny side of the glow-discharged (40 s) 300-mesh-Cu grids (Quantifoil) coated with a continuous carbon film and waited for 60 s to allow sample adsorption. Then, three drops of 20 μl, 20 μl, and 60 μl uranyl acetate (3%, v/v) stain solution were applied on the parafilm (Bemis). The grid was blotted from the side with a wedge of filter paper and washed with 4 μl of gel filtration buffer containing 20 mM Hepes (pH 7.0), 150 mM NaCl. Removing the excessive buffer and staining the grid with the third drop of uranyl acetate for 40 s followed by allowing it in the first two drops fastly. After wicking the stain away, the grid was allowed to air dry for 30 min and stored until imaging. For data collection, the grid was placed on a side-entry holder to load into a JEM-1400 and operated at 120 KV, condenser lens aperture 150 μm, spot size 1. And micrographs were collected using RADIUS software on a Morada G3 direct electron camera under magnification of ×150,000 (corresponding to a calibrated sampling of 2.56 Å per physical pixel). After further CTF estimation by CTFFIND4^52^, selected micrographs were subjected to EMAN2.31^53^ for neural network particle picking, with a threshold setting of −0.3 used to maximize inclusion of good particles. The resulting picked particles were extracted in Relion 4.0, with the box size as 128 pixels^54^. After two rounds of 2D classifications, the best classes selected by visual examination in cryoSPARC3.1^55^.

### Surface plasmon resonance (SPR) assay

The SPR assays were carried out using the Biacore 8K system with Biacore Insight Evaluation Software 3.0 (GE Healthcare) at room temperature with the CM5 sensor Chip (GE Healthcare). In brief, the chip was first immobilized with an anti-His antibody (Cytiva, 29234602, 1:20), which was further used to capture the monomeric peptidase domain of human ACE2-His at about 40 response units (RUs). Gradient concentrations of the RBD-HR/trimer protein ranging from 3.125 nM to 100 nM were then flew through the chip surface to record the real-time protein binding kinetics. For each sample, the test involves an association of 120 s and a dissociation of 300 s in the HBS-EP running buffer at a flow rate of 30 μl/min. Regeneration of the sensor chip was performed by running with the regeneration buffer (glycine pH 1.5) for 60 s. The association (ka) and dissociation (kd) rate constants, as well as the binding affinity value (KD), were determined.

### Analytical ultracentrifugation (AUC) assay

The AUC assay was carried out in a Beckman Coulter XL-I analytical ultracentrifuge using two-channel centerpieces. The RBD-HR/trimer protein was prepared in a buffer consisting of 20 mM Hepes pH 7.0 and 150 mM NaCl, loaded at the concentration of A280 = 0.8 absorbance units, and incubated at 4 °C for 1 h. Data were collected via absorbance detection at 18 °C with a rotor speed of 207,604 × *g*. The SV-AUC data were globally analyzed using the SEDFIT program and were fitted to a continuous c(s) distribution model to determine the molecular mass of the protein.

### The formulation of RBD-HR/trimer vaccine

MF59-like adjuvant was prepared as previously described^56^. The purified recombinant RBD-HR/trimer protein was diluted at the different concentrations (2–120 μg/ml) in PBS. The RBD-HR/trimer vaccine was formulated with different concentrations of RBD-HR/trimer protein and adjuvant at a volume ratio of 1:1.

### SARS-CoV-2 delta full-length S protein-encoding mRNA design and synthesis

The formulation of mRNA vaccine was prepared as previously described^57^. The Delta full-length S protein mRNA vaccine was designed to contain the several proline mutations in order to enhance its stability^58^. The nucleic acid sequence of Delta full-lengths S protein-encoding mRNA was integrated into the open reading frame of plasmid (hCD2.4) containing the T7 promoter and poly A tail, and was obtained by in vitro synthesis (Jiyu Technology Co., Ltd, Shenzhen, China), and was further capped to obtain in vitro transcribed (IVT) mRNA.

### Vaccinations of mice

Specific pathogen-free (SPF) female NIH mice (6–8 weeks) were purchased from HFK bioscience company (China) for immunization. Mice were maintained in a SPF animal facility (temperature: 21–25 °C; humidity: 30–70%; dark/light cycle: 12 h/12 h), and were adapted for at least one week before vaccination and immunized using a prime-boost regimen spaced 21 days apart. Mice (n = 6 per group) were intramuscularly injected with a dose range (1 μg, 5 μg, 10 μg or 20 μg) of RBD-HR/trimer protein formulated with adjuvants at a volume of 100 μl, respectively. As control, mice were injected with PBS, adjuvant or 10 μg RBD-HR/trimer protein alone. Blood samples were collected on day 7, 14, 56, and 100 after the first immunization, followed by centrifugation and serum collection.

For assay of heterologous third-dose booster, NIH mice were randomly divided into four groups. Three of the four groups were immunized with PBS, three doses of 5 μg mRNA/50 μl of encapsulated liposome (LPX)/Spike-mRNA, or 10 μg RBD-HR adjuvanted with MF59-like adjuvant, respectively, on day 0, 21, and 42. In the other group, 5 μg mRNA/50 μl of mRNA encapsulated liposome (LPX)/Spike-mRNA was intramuscularly injected to mice on day 0 and 21. On day 42, NIH mice in this group were immunized with 10 μg RBD-HR/trimer with adjuvant. Blood samples were collected from orbital veins of mice on day 84. Serum was centrifuged at 3500 × *g* for 10 min and stored at −20 °C before use. All animal experiments have been approved by the Institutional Animal Care and Use Committee of Sichuan University (Chengdu, Sichuan, China).

### Vaccinations of rats

Female Sprague-Dawley (SD) rats with an average weight of 180–200 g (7 weeks of age) were purchased from HFK bioscience company (China). The SD rats were randomized into five groups (*n* = 5) and immunized with PBS, low dose (15 μg), medium dose (30 μg), and high dose (60 μg) of RBD-HR/trimer vaccine on day 0, 21, and 42. The serum samples were collected on 56 after the first vaccination. All animal experiments have been approved by the Institutional Animal Care and Use Committee of Sichuan University (Chengdu, Sichuan, China).

### Enzyme-linked immunosorbent assays

Recombinant RBD proteins were dissolved in carbonate coating buffer (50 mM, pH 9.6) at 1 μg/ml, and were coated into 96-well plate (NUNC-MaxiSorp, Thermo Fisher Scientific) at 4 °C overnight for ELISA binding assay. Next day, each well was washed three times with PBS containing 0.1% Tween-20 (PBST), and was blocked with 100 μl PBST containing 1% BSA for 1 h at room temperature. Then, serum serially diluted were added and incubated for 1 h at 37 °C. After washing three times with PBST, horseradish peroxidase (HRP)‐conjugated anti‐mouse IgG antibodies (Invitrogen, 31430) were diluted at 1:10,000 with PBST and added to the wells (100 μl/well) to measure the titers of specific IgG. After incubation for 1 h at 37 °C, washed the plates five times with PBST and developed with 3,3′,5,5′-tetramethyl biphenyl diamine (TMB) for 10 min at room temperature. Stop the color development by adding 100 μl/well 1.0 M H_2_SO_4_. Finally, the absorbance was measured at 450 nm on a microplate reader (Spectramax ABS, Molecular Devices) with SoftMax Pro 7.1 software.

### Live SARS-CoV-2 virus neutralization assay

The titers of neutralizing antibodies in serum samples against the infection of ancestral SARS-CoV-2 and a variety of variants were determined by the authentic virus neutralization assay. Briefly, serum samples were diluted and co-incubated with live SARS-CoV-2 virus at 50% tissue-culture infectious dose (TCID_50_) at 37 °C for 1 h. Then, the mixture was added to Vero cells (ATCC CCL-81) (5 × 10^4^/well) and incubated for 3 days. The cytopathogenic effects (CPE) were recorded using the microscope, and the neutralizing titers of immunized serum that resulted in 50% neutralization were calculated.

### Pseudovirus neutralization assay

Different pseudoviruses were used for neutralization assay to detect the neutralizing antibodies in sera from immunized mice. The wildtype and variants of SARS-CoV-2 luciferase-expressing pseudoviruses were purchased from Genomeditech (China), and single L452R/T478K mutation or the double L452R + T478K substitutions pseudoviruses were purchased from Vazyme company. Briefly, serum samples from each group were threefold serial dilutions ranging from 90 to 196,830 and co-incubated with 50 μl diluted pseudovirus for 1 h at 37 °C. Subsequently, added 293T/ACE2 cells (1.2 × 10^4^/well) and incubated for 48 h to express the luciferase. Finally, removed the cell supernatant, added 100 µl lysis reagent with luciferase substrate from a luciferase kit (Promega, USA), and detected relative light unit (RLU) by a multi-mode microplate reader (PerkinElmer, USA) with Kaleido 3.0 software.

The percentage of neutralization was calculated by the RLU measured in the pseudovirus control well (pseudovirus and cells) subtracting the RLU measured in the sample well (sample, virus, and cells) and dividing the RLU of the pseudovirus control well subtracting the cell control well (cells alone), and multiplying by 100%. Pseudovirus 50% neutralizing titers were expressed as the highest dilution that caused 50% inhibitory relative to the average of the virus control wells and calculated by a non-linear regression model (inhibitor versus normalized response) in GraphPad Prism.

### Blockade of RBD binding to ACE2 receptor

Three RBD-Fc fusion proteins including wild-type RBD-Prototype, RBD-Delta, RBD-Omicron were used to detect the binding to ACE2 receptor in the absence or presence of sera, respectively. In brief, dissolving the RBD-Fc protein at 0.4 μg/ml in PBS supplemented with 1% BSA (BPBS). Mice sera with serially dilution were added to the RBD-Fc protein solution and incubated at room temperature for 30 min. Then, added the mixture to the 293T/ACE2 cells and incubated at room temperature. After 30 min, cells were washed three times with BPBS to remove unbound proteins. Then, added the PE-conjugated anti-human IgG Fc antibodies (BioLegend, USA) at 1:100 to stain at 4 °C for 30 min. The cells were detected by the NovoCyte Flow Cytometer (ACEA Biosciences) and the binding assay was analyzed with NovoExpress 1.4.1 software.

### Cell–cell fusion assay

HEK-293T cells co-transfected with a EGFP encoding plasmid (pEGFP-C1) and a vector encoding SARS-CoV-2 S glycoprotein with truncated C-terminal 18aa were used as the effector cells (293T/EGFP/S). And 293T cells stably expressing human ACE2 receptor were utilized as target cells (293T/ACE2). A total of 5000 per well effector cells (293T/EGFP/S) were incubated with immunized mouse serum at 37 °C for 4 h. Then, 5000 per well target cells (293T/ACE2) were added and co-cultured with effector cells at 37 °C for 4 h. The HEK-293T cells only transfected with EGFP encoding plasmid (293T/EGFP) incubated with target cells (293T/ACE2) cells were used as the negative control. After cells were stained with Hoechst (Beyotime biotechnology, Hoechst 33342) at 37 °C for 10 min, the syncytium formation was observed by an inverted fluorescence microscope (Olympus Corporation) and images were collected by CellSens Standard 2.1 software. The fusion ratio was calculated by observing the number of fused and unfused cells at five fields randomly.

### Antigenicity analyses of spike RBD sequence

The amino acid sequences of prototype (with L452 and T478) and Delta-variant (with R452 and K478) RBDs were analyzed with Lasergene and the antigenicity index for each residue was calculated using the Jameson-Wolf method^33^. The index values of L452, R452, T478, and K478 were individually recorded and pairwise compared.

### Flow cytometry

Immunized NIH mice were sacrificed on day 49 after the first immunization. Lymphocytes from spleen were isolated to investigate T cellular immune responses. T lymphocytes in spleen were stained at 4 °C for 30 min with following antibodies at 1:100 dilution: PerCP/Cyanine5.5-conjugated anti-mouse CD3 (BioLegend, 100718), APC-conjugated anti-mouse CD4 (BioLegend, 100412), FITC-conjugated anti-mouse CD8 (BioLegend, 100706), Brilliant Violet 510-conjugated anti-mouse CD44 (BioLegend, 103044), PE-conjugated anti-mouse CD62L (BioLegend, 161204). For assay of the formation of germinal centers (GC) in spleens, T follicular helper (Tfh) cells were stained with PerCP/Cyanine5.5-conjugated anti-mouse CD3, FITC-conjugated anti-mouse CD4 (BioLegend, 100406), PE-conjugated anti-mouse CD19 (BioLegend, 553786), APC-conjugated anti-mouse CXCR5 (BioLegend, 145506), and Brilliant Violet 421-conjugated anti-mouse PD-1 antibodies (BioLegend, 135218). GC B cells were stained with PerCP/Cyanine5.5-conjugated anti-mouse CD3, Brilliant Violet 421-conjugated anti-mouse CD19 (BioLegend, 115538), FITC-conjugated anti-mouse GL7 (BioLegend, 144612), and PE-CF594-conjugated anti-mouse CD95 antibodies (BioLegend, 582499).

For intracellular cytokine staining (ICS), lymphocytes from spleen were isolated at a sterile condition and cultured in complete 1640 medium supplied with 10% FBS, 100 μg/ml streptomycin, 100 U/ml penicillin, 1 mM pyruvate (all from Gibco, USA), 50 μM β-mercaptoethanol, and 20 U/ml IL-2 (all from Sigma-Aldrich, USA). 10 μg/ml RBD was added to activate cells. Before cell staining, brefeldin A (BFA, BD Biosciences) were used to block intracellular cytokine secretion. Culture supernatants were collected to measure the levels of IL-4 and IFN-γ by ELISA. Cells were stained with PerCP/Cyanine5.5-conjugated anti-mouse CD45R (BioLegend, 103236), PE-Cy7-conjugated anti-mouse MHCII (BioLegend, 107630), APC-conjugated anti-mouse CD4, FITC-conjugated anti-mouse CD8, Brilliant Violet 510-conjugated anti-mouse CD44 antibodies for 30 min at 4 °C. Then, cells were fixed and permeabilized, and stained with PE-conjugated anti-mouse IFN-γ (BioLegend, 554412) and Brilliant Violet 421 anti-mouse IL-4 antibodies (BioLegend, 504120) at room temperature for 1 h. Cells were detected by the NovoCyte Flow Cytometer (ACEA Biosciences) and data were analyzed with NovoExpress 1.4.1 software.

### Challenge of SARS-CoV-2 Omicron variant in hACE2 mice

Transgenic hACE2 (6–8 weeks) with ICR background were also divided into four groups (*n* = 6): (1) low dose (10 μg) of RBD-HR/trimer with adjuvant, (2) high dose (20 μg) of RBD-HR/trimer with adjuvant, (3) adjuvant alone, and (4) PBS. We adopted a prime-boost regimen spaced 21 days apart with three injections. Transgenic hACE2 mice were challenged with 1 × 10^5^ PFU of SARS-CoV-2 Omicron variant via intranasal route. On day 4 post challenge, the mice were euthanized for serum collection and tissue processing. The nasal turbinates, trachea, and lung tissue were collected for assay of viral loads and histological examination. Viral genomic RNA (gRNA) was measured by reverse-transcription quantitative polymerase chain reaction (RT-qPCR), using the following primer and probe sequences (forward, 5′-GACCCCAAAATCAGCGAAAT-3′; reverse, 5′-TCTGGTTACTGCCAGTTGAATCTG-3′; probe, 5′-FAM-ACGCCGCATTACGTTTGGTGGACC-BHQ1-3′). All procedures associated with challenge of SARS-CoV-2 in mice study were reviewed and approved by the Institutional Animal Care and Use Committee of the Institute of Medical Biology, Chinese Academy of Medical Sciences, and performed in the ABSL-4 facility of Kunming National High-level Biosafety Primate Research Center.

### Challenge of SARS-CoV-2 Delta variant in a non-human primate model

Twelve male non-human primates (2–4 years old) were assigned to four groups: (1) non-human primates in high-dose group were immunized with 60 μg RBD-HR/trimer protein with adjuvant for 0.5 ml volume (*n* = 4); (2) non-human primates in low-dose group were immunized with 30 μg RBD-HR/trimer protein with adjuvant for 0.25 ml volume (*n* = 3); (3) animals in PBS group were injected 0.5 ml PBS as control (*n* = 3), and (4) adjuvant group were treated with 0.5 ml MF59-like adjuvant alone (*n* = 2). All animals were immunized intramuscularly with three injections spaced 21 days apart before Delta variants challenge. The immunized non-human primates were sedated and then challenged with 1 × 10^6^ PFU of SARS-CoV-2 Delta virus via intranasal (0.5 ml) and intratracheal (0.5 ml) routes on day 75 after the first immunization. Blood samples were collected on day 0, 21, 42, 56, 75 (before challenge) and 1, 3, 5, 7 days post infection (dpi) to detect functional antibodies, routine blood tests, blood chemistry and hematology. All non-human primates were euthanized on 7 days post infection for tissue processing. Viral genomic RNA (gRNA) and sub-genomic RNA (sgRNA) in lung tissue were measured by reverse-transcription quantitative polymerase chain reaction (RT-qPCR). For histological examination, tissues were fixed with 10% neutral-buffered formalin, embedded in paraffin, and sections were stained with hematoxylin and eosin (H&E). Pathologic slides were digitized using ZEN blue 2.3 (Carl Zeiss AG). Approval for the live SARS-CoV-2 challenge in non-human primate study was provided by the Institutional Animal Care and Use Committee of the Institute of Medical Biology, Chinese Academy of Medical Sciences, and performed in the ABSL-4 facility of Kunming National High-level Biosafety Primate Research Center.

### Statistics and reproducibility

All experiments except the SARS-CoV-2 challenge experiment in animals were repeated independently at least twice with similar results. Statistical analyses were performed using Prism 8.0 (GraphPad software). The *P* values were determined using One- or Two-way analysis of variance (ANOVA) as indicated in each figure legend. *P* values < 0.05 were considered significant, n.s., not significant.

### Reporting summary

Further information on research design is available in the Nature Research Reporting Summary linked to this article.

## Supplementary information

Supplementary Information

Supplementary Dataset 1

Reporting Summary

## Data Availability

All data that support the findings of this study are available upon request from the corresponding author. Source data are provided with this paper.
