# Supplementary material for: A self-assembled trimeric protein vaccine induces protective immunity against Omicron variant
Source: Nat Commun. 2022 Sep 17;13:5459. doi: 10.1038/s41467-022-33209-9 (PMC9482656; doi:10.1038/s41467-022-33209-9)
Supplement: Supplementary file 1 — Supplementary Information [file 41467_2022_33209_MOESM1_ESM.pdf]

Supplementary Information

A self-assembled trimeric protein vaccine induces protective immunity against Omicron variant

Contents:

Supplementary Figure 1, 2, 3, 4

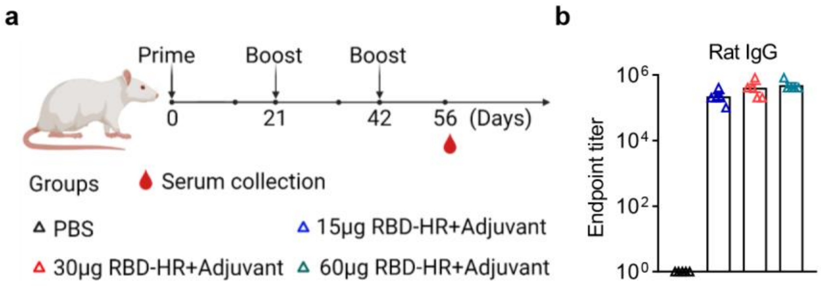

Supplementary Fig. 1. RBD-HR/trimer induced sustained humoral immune response in rats. a, The schematic representation of the rat immunization and sample collection protocol. Sprague-Dawley (SD) rats were immunized on day 0, 21, 42 with intramuscular injections of 15 μg, 30 μg or 60 μg of RBD-HR vaccine, respectively. Serum samples were collected on day 56 after the first vaccination. **b,** The end point titers of anti-RBD specific IgG in rat sera induced by RBD-HR vaccine on day 56 were performed by ELISA (n = 5 rats per group). Data are presented as geometric mean values +/- SD.

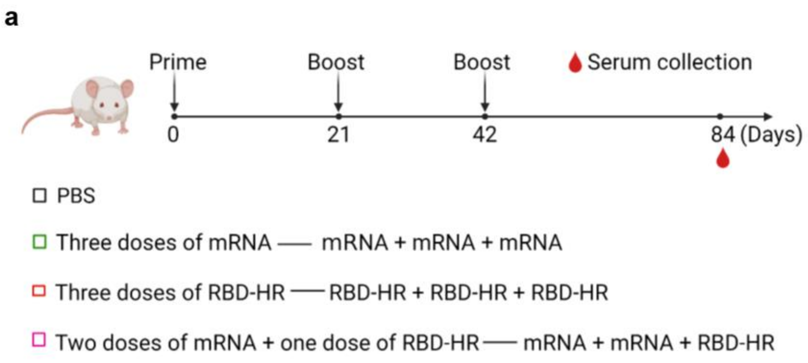

Supplementary Fig. 2. The schematic representation of the homologous and heterologous vaccination. a, Three doses of mRNA vaccine: the mice were immunized with three doses of 5 μg mRNA/50 μL of encapsulated liposome (LPX)/Spike-mRNA; three doses of RBD-HR/trimer vaccine: the mice were immunized with three doses of 10 μg RBD-HR/trimer vaccine; two doses of mRNA and one dose of RBD-HR/trimer: the mice were immunized with two doses of 5 μg mRNA/50 μL of encapsulated liposome (LPX)/Spike-mRNA, and subsequently immunized with one dose of RBD-HR/trimer vaccine. And a prime-boost regimen spaced 21 days apart was adopted.

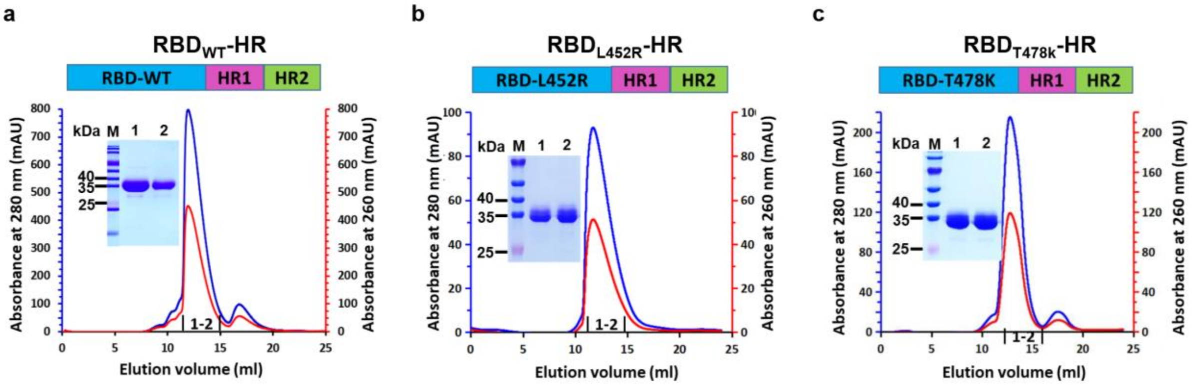

Supplementary Fig. 3. Successful expression of $\mathbf{RBD_{WT}}$-HR, $\mathbf{RBD_{L452R}}$-HR and $\mathbf{RBD_{T478K}}$-HR proteins. a-c, Representative elution chromatographs of the recombinant RBD_{WT}-HR (a), RBD_{L452R}-HR (b) and RBD_{T478K}-HR (c) proteins using a calibrated Superdex 200 Increase column. The SDS-PAGE analysis of the eluted protein was shown.

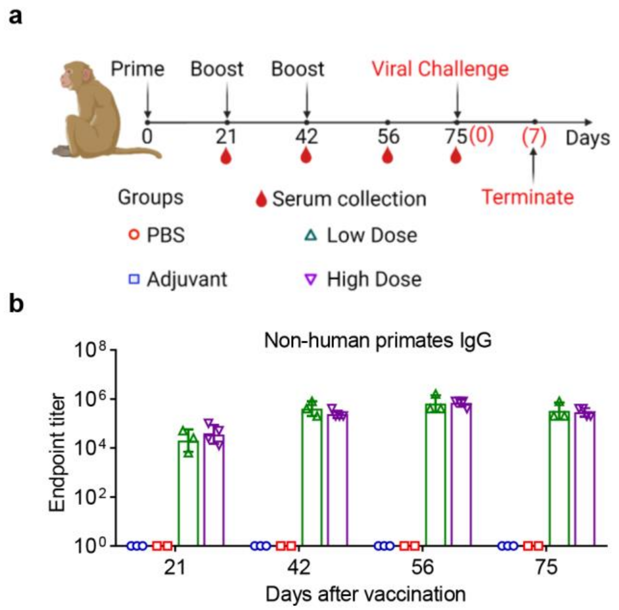

Supplementary Fig. 4. RBD-HR/trimer induced sustained humoral immune response in non-human primates. a, Non-human primates were immunized on day 0, 21, 42 with intramuscular injections of 30 μg (low dose) or 60 μg (high dose) of RBD-HR/trimer vaccine, respectively. Non-human primates treated with PBS or adjuvant were used as control. The immunized non-human primates were challenged with 1×10^6 PFU of SARS-CoV-2 Delta virus on day 75 after the first immunization. **b,** The end point titers of anti-RBD specific binding antibodies induced by RBD-HR/trimer vaccine were performed, PBS: n = 3, Adjuvant: n = 2, Low dose group: n = 3, High dose: n = 4 non-human primates. Data are presented as geometric mean values +/- SD.
