# Supplementary material for: A self-assembled trimeric protein vaccine induces protective immunity against Omicron variant
Source: Nat Commun. 2022 Sep 17;13:5459. doi: 10.1038/s41467-022-33209-9 (PMC9482656; doi:10.1038/s41467-022-33209-9)
Supplement: Supplementary file 3 — Reporting Summary [file 41467_2022_33209_MOESM3_ESM.pdf]

nature research

Corresponding author(s): Xiawei Wei

Last updated by author(s): Sep 2, 2022

Reporting Summary

Nature Research wishes to improve the reproducibility of the work that we publish. This form provides structure for consistency and transparency in reporting. For further information on Nature Research policies, see our Editorial Policies and the Editorial Policy Checklist.

Statistics

For all statistical analyses, confirm that the following items are present in the figure legend, table legend, main text, or Methods section.

n/a	Confirmed	
☐	☒	The exact sample size (*n*) for each experimental group/condition, given as a discrete number and unit of measurement
☐	☒	A statement on whether measurements were taken from distinct samples or whether the same sample was measured repeatedly
☐	☒	The statistical test(s) used AND whether they are one- or two-sided<br>*Only common tests should be described solely by name; describe more complex techniques in the Methods section.*
☒	☐	A description of all covariates tested
☐	☒	A description of any assumptions or corrections, such as tests of normality and adjustment for multiple comparisons
☐	☒	A full description of the statistical parameters including central tendency (e.g. means) or other basic estimates (e.g. regression coefficient) AND variation (e.g. standard deviation) or associated estimates of uncertainty (e.g. confidence intervals)
☐	☒	For null hypothesis testing, the test statistic (e.g. *F*, *t*, *r*) with confidence intervals, effect sizes, degrees of freedom and *P* value noted<br>*Give P values as exact values whenever suitable.*
☒	☐	For Bayesian analysis, information on the choice of priors and Markov chain Monte Carlo settings
☒	☐	For hierarchical and complex designs, identification of the appropriate level for tests and full reporting of outcomes
☒	☐	Estimates of effect sizes (e.g. Cohen's *d*, Pearson's *r*), indicating how they were calculated

Our web collection on statistics for biologists contains articles on many of the points above.

Software and code

Policy information about availability of computer code

Data collection	1. Affinity and kinetics data for RBD-HR were collected using Biacore 8K by GE Healthcare with Biacore Insight Evaluation Software 3.0 (General Electric Company)<br>2. Flow cytometric data were collected using NovoCyte with NovoExpress 1.4.1 (ACEA bioscience. Inc).<br>3. Firefly luciferase assay data were collected using multi-mode microplate reader with kaleido 3.0 (PerkinElmer).<br>4. Fluorescent images were obtained using Olympus IX73 microscope with CellSens Standard software 2.1 (Olympus Corporation).<br>5. Enzyme linked Immunosorbent assay data were collected using Spectramax ABS with SoftMax Pro 7.1 (Molecular Devices).<br>6. Pathologic slides were digitized using ZEN blue 2.3 (Carl Zeiss AG).<br>7. Western blot data were collected using Clinx ChemiScope Series with ChemiCapturePAD V2018.5 (Clinx Science Instrument Co., Ltd).
Data analysis	The statistical analyses were performed with GraphPad Prism version 8.0 for One- or Two-way ANOVA test. Flow cytometry data analyzed by NovoExpress 1.4.1. Fluorescent images and Blots were analyzed by Adobe Photoshop 2020 v21.0.2.57.

For manuscripts utilizing custom algorithms or software that are central to the research but not yet described in published literature, software must be made available to editors and reviewers. We strongly encourage code deposition in a community repository (e.g. GitHub). See the Nature Research guidelines for submitting code & software for further information.

Data

Policy information about availability of data

All manuscripts must include a data availability statement. This statement should provide the following information, where applicable:

- Accession codes, unique identifiers, or web links for publicly available datasets
- A list of figures that have associated raw data
- A description of any restrictions on data availability

All data that support the findings of this study are available upon request from the corresponding author. Source data are provided with this paper.

Field-specific reporting

Please select the one below that is the best fit for your research. If you are not sure, read the appropriate sections before making your selection.

☒ Life sciences ☐ Behavioural & social sciences ☐ Ecological, evolutionary & environmental sciences

For a reference copy of the document with all sections, see nature.com/documents/nr-reporting-summary-flat.pdf

Life sciences study design

All studies must disclose on these points even when the disclosure is negative.

Sample size	No statistical methods were used to determine sample size.
Data exclusions	No data were excluded from the analysis.
Replication	Most of experiments were repeated with at least two biologically independent for all results presented in the manuscript, and the replicate experiments were successful.
Randomization	Mice, rats and non-human primates were randomly assigned to different treatment groups.
Blinding	For animal immunization, technicians were not blinded to group allocation as they need to know the identity document of the corresponding vaccinated animal. For date collection and analysis of other in vivo and in vitro experiment, the investigators were blinded to group allocations.

Behavioural & social sciences study design

All studies must disclose on these points even when the disclosure is negative.

Study description	*Briefly describe the study type including whether data are quantitative, qualitative, or mixed-methods (e.g. qualitative cross-sectional, quantitative experimental, mixed-methods case study).*
Research sample	*State the research sample (e.g. Harvard university undergraduates, villagers in rural India) and provide relevant demographic information (e.g. age, sex) and indicate whether the sample is representative. Provide a rationale for the study sample chosen. For studies involving existing datasets, please describe the dataset and source.*
Sampling strategy	*Describe the sampling procedure (e.g. random, snowball, stratified, convenience). Describe the statistical methods that were used to predetermine sample size OR if no sample-size calculation was performed, describe how sample sizes were chosen and provide a rationale for why these sample sizes are sufficient. For qualitative data, please indicate whether data saturation was considered, and what criteria were used to decide that no further sampling was needed.*
Data collection	*Provide details about the data collection procedure, including the instruments or devices used to record the data (e.g. pen and paper, computer, eye tracker, video or audio equipment) whether anyone was present besides the participant(s) and the researcher, and whether the researcher was blind to experimental condition and/or the study hypothesis during data collection.*
Timing	*Indicate the start and stop dates of data collection. If there is a gap between collection periods, state the dates for each sample cohort.*
Data exclusions	*If no data were excluded from the analyses, state so OR if data were excluded, provide the exact number of exclusions and the rationale behind them, indicating whether exclusion criteria were pre-established.*
Non-participation	*State how many participants dropped out/declined participation and the reason(s) given OR provide response rate OR state that no participants dropped out/declined participation.*
Randomization	*If participants were not allocated into experimental groups, state so OR describe how participants were allocated to groups, and if allocation was not random, describe how covariates were controlled.*

Ecological, evolutionary & environmental sciences study design

All studies must disclose on these points even when the disclosure is negative.

Study description	*Briefly describe the study. For quantitative data include treatment factors and interactions, design structure (e.g. factorial, nested, hierarchical), nature and number of experimental units and replicates.*
Research sample	*Describe the research sample (e.g. a group of tagged Passer domesticus, all Stenocereus thurberi within Organ Pipe Cactus National Monument), and provide a rationale for the sample choice. When relevant, describe the organism taxa, source, sex, age range and any manipulations. State what population the sample is meant to represent when applicable. For studies involving existing datasets, describe the data and its source.*
Sampling strategy	*Note the sampling procedure. Describe the statistical methods that were used to predetermine sample size OR if no sample-size calculation was performed, describe how sample sizes were chosen and provide a rationale for why these sample sizes are sufficient.*
Data collection	*Describe the data collection procedure, including who recorded the data and how.*
Timing and spatial scale	*Indicate the start and stop dates of data collection, noting the frequency and periodicity of sampling and providing a rationale for these choices. If there is a gap between collection periods, state the dates for each sample cohort. Specify the spatial scale from which the data are taken*
Data exclusions	*If no data were excluded from the analyses, state so OR if data were excluded, describe the exclusions and the rationale behind them, indicating whether exclusion criteria were pre-established.*
Reproducibility	*Describe the measures taken to verify the reproducibility of experimental findings. For each experiment, note whether any attempts to repeat the experiment failed OR state that all attempts to repeat the experiment were successful.*
Randomization	*Describe how samples/organisms/participants were allocated into groups. If allocation was not random, describe how covariates were controlled. If this is not relevant to your study, explain why.*
Blinding	*Describe the extent of blinding used during data acquisition and analysis. If blinding was not possible, describe why OR explain why blinding was not relevant to your study.*

Did the study involve field work? ☐ Yes ☐ No

Field work, collection and transport

Field conditions	*Describe the study conditions for field work, providing relevant parameters (e.g. temperature, rainfall).*
Location	*State the location of the sampling or experiment, providing relevant parameters (e.g. latitude and longitude, elevation, water depth).*
Access & import/export	*Describe the efforts you have made to access habitats and to collect and import/export your samples in a responsible manner and in compliance with local, national and international laws, noting any permits that were obtained (give the name of the issuing authority, the date of issue, and any identifying information).*
Disturbance	*Describe any disturbance caused by the study and how it was minimized.*

Reporting for specific materials, systems and methods

We require information from authors about some types of materials, experimental systems and methods used in many studies. Here, indicate whether each material, system or method listed is relevant to your study. If you are not sure if a list item applies to your research, read the appropriate section before selecting a response.

Materials & experimental systems

n/a	Involved in the study	
☐	☒	Antibodies
☐	☒	Eukaryotic cell lines
☒	☐	Palaeontology and archaeology
☐	☒	Animals and other organisms
☒	☐	Human research participants
☒	☐	Clinical data
☒	☐	Dual use research of concern

Methods

n/a	Involved in the study	
☒	☐	ChIP-seq
☐	☒	Flow cytometry
☒	☐	MRI-based neuroimaging

Antibodies

Antibodies used	The following antibodies were used at 1:100 dilution for flow cytometry .<br>PE-Cy7-conjugated anti-mouse MHC-II antibody: Provide supplier name: BioLegend, Cat: 107630, Clone: M5/114.15.2, Lot: B199459.<br>PerCP/Cy5.5-conjugated anti-mouse CD45R antibody: Provide supplier name: BioLegend, Cat: 103236, Clone: RA3-6B2, Lot: B295257.<br>APC-conjugated anti-mouse CD4 antibody: Provide supplier name: BioLegend, Cat: 100412, Clone: GK1.5, Lot: B293107.<br>FITC-conjugated anti-mouse CD8 antibody: Provide supplier name: BioLegend, Cat: 100706, Clone: 53-6.7, Lot: B278606.<br>Brilliant Violet 510™-conjugated anti-mouse CD44 antibody: Provide supplier name: BioLegend, Cat: 103044, Clone: IM7, Lot: B298812.<br>PE-conjugated anti-mouse IFN-γ antibody: Provide supplier name: BD Bioscience, Cat: 554412, Clone: XMG1.2, Lot: 3217951.<br>Brilliant Violet 421™-conjugated anti-mouse IL-4 antibody: Provide supplier name: BioLegend, Cat: 504120, Clone: 11B11, Lot: B282852.<br>PE-conjugated anti-mouse CD62L antibody: Provide supplier name: BD Bioscience, Cat: 161204, Clone: W18021D, Lot: B359280.<br>PerCP/Cy5.5-conjugated anti-mouse CD3 antibody: Provide supplier name: BioLegend, Cat: 100718, Clone: 17A2, Lot: B351062.<br>PE-conjugated anti-mouse CD19 antibody: Provide supplier name: BioLegend, Cat: 553786, Clone: 1D3, Lot: B239139.<br>FITC-conjugated anti-mouse CD4 antibody: Provide supplier name: BioLegend, Cat: 100406, Clone: GK1.5, Lot: B367824.<br>APC-conjugated anti-mouse CXCR5 antibody: Provide supplier name: BioLegend, Cat: 145506, Clone: L138D7, Lot: B355148.<br>Brilliant Violet 421™-conjugated anti-mouse PD-1 antibody: Provide supplier name: BioLegend, Cat: 135218, Clone: 29F.1A12, Lot: B349792.<br>Brilliant Violet 421™-conjugated anti-mouse CD19 antibody: Provide supplier name: BioLegend, Cat: 115538, Clone: 6D5, Lot: B344438.<br>FITC-conjugated anti-mouse GL7 antibody: Provide supplier name: BioLegend, Cat: 144612, Clone: GL7, Lot: B180155.<br>PE-CF594-conjugated anti-mouse CD95 antibody: Provide supplier name: BioLegend, Cat: 582499, Clone: J02, Lot: 4134891.<br>PE-conjugated anti-human-IgG Fc antibody: Provide supplier name: BioLegend, Cat:410708, Clone: M1310G05, Lot:B338536.<br><br>The following antibodies were used at indicated dilutions for surface plasmon resonance assay, enzyme-linked immunosorbent assay and western blot.<br>Anti-His antibodies: Provide supplier name: Cytiva, Cat: 29234602, 1:20 dilution.<br>HRP-conjugated Mouse IgG antibody: Provide supplier name: Invitrogen, Cat: 31430, Lot: WA319701, 1:10000 dilution.<br>Mouse SARS-CoV-2 (2019-nCoV) Spike antibody: Provide supplier name:Sino Biological Inc, Cat:40591-MM42; 1:2000 dilution.
Validation	All the antibodies are validated for the use of flow cytometry, surface plasmon resonance, enzyme-linked immunosorbent assays and western bolt.<br>PE-Cy7-conjugated anti-mouse MHC-II antibody, PerCP/Cy5.5-conjugated anti-mouse CD45R antibody, APC-conjugated anti-mouse CD4 antibody, FITC-conjugated anti-mouse CD8 antibody, Brilliant Violet 510™-conjugated anti-mouse CD44 antibody, PE-conjugated anti-mouse IFN-γ antibody, Brilliant Violet 421™-conjugated anti-mouse IL-4 antibody, PE-conjugated anti-mouse CD62L antibody, PerCP/Cy5.5-conjugated anti-mouse CD3 antibody, PE-conjugated anti-mouse CD19 Antibody, FITC-conjugated anti-mouse CD4 antibody, APC-conjugated anti-mouse CXCR5 antibody, Brilliant Violet 421™-conjugated anti-mouse PD-1 antibody, Brilliant Violet 421™-conjugated anti-mouse CD19 antibody, FITC-conjugated anti-mouse GL7 antibody and PE-CF594-conjugated anti-mouse CD95 antibody used in flow cytometry of spleen organoids.<br>PE-conjugated anti-human-IgG Fc antibody used in flow cytometry of the binding of RBD to 293T/ACE2 cells.<br>HRP-conjugated Mouse IgG antibody used in ELISA binding assay.<br>Mouse SARS-CoV-2 (2019-nCoV) Spike antibody used in western bolt.

Eukaryotic cell lines

Policy information about cell lines

Cell line source(s)	HEK293T cells (ATCC CRL-11268), Sf9 cells (ATCC CRL-1711), Vero cells (ATCC CCL-81).
Authentication	No authentication has been used.
Mycoplasma contamination	Neither of the cell lines used in this study tested positive for Mycoplasma.
Commonly misidentified lines (See ICLAC register)	No commonly misidentified lines were used.

Palaeontology and Archaeology

Specimen provenance	*Provide provenance information for specimens and describe permits that were obtained for the work (including the name of the issuing authority, the date of issue, and any identifying information).*
Specimen deposition	*Indicate where the specimens have been deposited to permit free access by other researchers.*
Dating methods	*If new dates are provided, describe how they were obtained (e.g. collection, storage, sample pretreatment and measurement), where they were obtained (i.e. lab name), the calibration program and the protocol for quality assurance OR state that no new dates are provided.*

☐ Tick this box to confirm that the raw and calibrated dates are available in the paper or in Supplementary Information.

Ethics oversight	*Identify the organization(s) that approved or provided guidance on the study protocol, OR state that no ethical approval or guidance was required and explain why not.*

Note that full information on the approval of the study protocol must also be provided in the manuscript.

Animals and other organisms

Policy information about studies involving animals; ARRIVE guidelines recommended for reporting animal research

Laboratory animals	Female NIH mice (6-8 weeks) and transgenic hACE2 mice (6-8 weeks) with ICR background were provided by the HFK bioscience company (Beijing, China) and were maintained in a SPF animal facility (temperature: 21-25°C ?? ; humidity: 30-70 %; dark/light cycle: 12h/12h) in Sichuan University.<br><br>Female Sprague-Dawley (SD) rats (7 weeks) were provided by the HFK bioscience company (Beijing, China).<br><br>Male non-human primates (Macaca mulatta) (2-4 years old) for the immunization and challenge provided by National Kunming High-level Biosafety Primate Research Center, Institute of Medical Biology, Chinese Academy of Medical Sciences and Peking Union Medical College, Yunnan China.
Wild animals	No wild animals were used in this study.
Field-collected samples	No field-collected samples were used in the study.
Ethics oversight	All animal studies carried out were approved by the Animal Care and Use Committee of Sichuan University (Chengdu, Sichuan, China). All procedures involved in the immunization and challenge in the non-human primates and transgenic hACE2 mice study were reviewed and approved by the Institutional Animal Care and Use Committee of Institute of Medical Biology, Chinese Academy of Medical Science, and performed in the ABSL-4 facility of Kunming National High-level Biosafety Primate Research Center, Yunnan, China.

Note that full information on the approval of the study protocol must also be provided in the manuscript.

Human research participants

Policy information about studies involving human research participants

Population characteristics	*Describe the covariate-relevant population characteristics of the human research participants (e.g. age, gender, genotypic information, past and current diagnosis and treatment categories). If you filled out the behavioural & social sciences study design questions and have nothing to add here, write "See above."*
Recruitment	*Describe how participants were recruited. Outline any potential self-selection bias or other biases that may be present and how these are likely to impact results.*
Ethics oversight	*Identify the organization(s) that approved the study protocol.*

Note that full information on the approval of the study protocol must also be provided in the manuscript.

Clinical data

Policy information about clinical studies
All manuscripts should comply with the ICMJE guidelines for publication of clinical research and a completed CONSORT checklist must be included with all submissions.

Clinical trial registration	*Provide the trial registration number from ClinicalTrials.gov or an equivalent agency.*
Study protocol	*Note where the full trial protocol can be accessed OR if not available, explain why.*
Data collection	*Describe the settings and locales of data collection, noting the time periods of recruitment and data collection.*
Outcomes	*Describe how you pre-defined primary and secondary outcome measures and how you assessed these measures.*

Dual use research of concern

Policy information about dual use research of concern

Hazards

Could the accidental, deliberate or reckless misuse of agents or technologies generated in the work, or the application of information presented in the manuscript, pose a threat to:

No | Yes

- [ ] [ ] Public health
- [ ] [ ] National security
- [ ] [ ] Crops and/or livestock
- [ ] [ ] Ecosystems
- [ ] [ ] Any other significant area

Experiments of concern

Does the work involve any of these experiments of concern:

No | Yes

- [ ] [ ] Demonstrate how to render a vaccine ineffective
- [ ] [ ] Confer resistance to therapeutically useful antibiotics or antiviral agents
- [ ] [ ] Enhance the virulence of a pathogen or render a nonpathogen virulent
- [ ] [ ] Increase transmissibility of a pathogen
- [ ] [ ] Alter the host range of a pathogen
- [ ] [ ] Enable evasion of diagnostic/detection modalities
- [ ] [ ] Enable the weaponization of a biological agent or toxin
- [ ] [ ] Any other potentially harmful combination of experiments and agents

ChIP-seq

Data deposition

- [ ] Confirm that both raw and final processed data have been deposited in a public database such as GEO.
- [ ] Confirm that you have deposited or provided access to graph files (e.g. BED files) for the called peaks.

Data access links
May remain private before publication.
For "Initial submission" or "Revised version" documents, provide reviewer access links. For your "Final submission" document, provide a link to the deposited data.

Files in database submission
Provide a list of all files available in the database submission.

Genome browser session
(e.g. UCSC)
Provide a link to an anonymized genome browser session for "Initial submission" and "Revised version" documents only, to enable peer review. Write "no longer applicable" for "Final submission" documents.

Methodology

Replicates
Describe the experimental replicates, specifying number, type and replicate agreement.

Sequencing depth
Describe the sequencing depth for each experiment, providing the total number of reads, uniquely mapped reads, length of reads and whether they were paired- or single-end.

Antibodies
Describe the antibodies used for the ChIP-seq experiments; as applicable, provide supplier name, catalog number, clone name, and lot number.

Peak calling parameters
Specify the command line program and parameters used for read mapping and peak calling, including the ChIP, control and index files used.

Data quality
Describe the methods used to ensure data quality in full detail, including how many peaks are at FDR 5% and above 5-fold enrichment.

Software
Describe the software used to collect and analyze the ChIP-seq data. For custom code that has been deposited into a community repository, provide accession details.

Flow Cytometry

Plots

Confirm that:

☒ The axis labels state the marker and fluorochrome used (e.g. CD4-FITC).

☒ The axis scales are clearly visible. Include numbers along axes only for bottom left plot of group (a 'group' is an analysis of identical markers).

☒ All plots are contour plots with outliers or pseudocolor plots.

☒ A numerical value for number of cells or percentage (with statistics) is provided.

Methodology

Sample preparation

Splenocytes activation
Lymphocytes from spleen and inguinal lymph nodes were isolated to investigate T cellular immune responses. lymphocytes from spleen were isolated at a sterile condition and cultured in complete 1640 medium supplied with 10% FBS, 100 μg/ml streptomycin, 100 U/ml penicillin, 1 mM pyruvate (all from Gibco, USA), 50 μM β-mercaptoethanol, and 20 U/ml IL-2 (all from Sigma-Aldrich, USA). 10 μg/ml RBD was added to activate cells. Before cell staining, brefeldin A (BFA, BD Biosciences) were used to block intracellular cytokine secretion. Culture supernatants were collected to measure the levels of IL-4 and IFN-γ by ELISA. Cells were stained with PerCP/Cyanine5.5-conjugated ant-mouse CD45R, PE-Cy7-conjugated anti-mouse MHC II, APC-conjugated anti-mouse CD4, FITC--conjugated anti-mouse CD8, Brilliant Violet 510-conjugated anti-mouse CD44 antibodies for 30 min at 4 °C. Then, cells were fixed and permeabilized, and stained with PE-conjugated anti-mouse IFN-γ and Brilliant Violet 421 anti-mouse IL-4 antibodies at room temperature for 1 hour.

Blockade of RBD binding to ACE2 receptor
The assay for blockade of RBD-Fc binding to surface ACE2 receptor was performed as described previously. Three RBD-Fc fusion proteins including wildtype RBD-Prototype, RBD-Delta, RBD-Omicron were used to detect the binding to ACE2 receptor in the absence or presence of serum, respectively. In brief, dissolving the RBD-Fc protein at 0.4 μg/ml in PBS supplemented with 1% BSA (BPBS). Mice sera with serially dilution were added to the RBD-Fc protein solution and incubated at room temperature for 30 min. Then, added the mixture to the 293T/ACE2 cells and incubated at room temperature. After 30 min, cells were washed three times with BPBS to remove unbound proteins. Then, added the PE-conjugated anti-human IgG Fc antibodies (BioLegend, USA) at 1:1000 to stain at 4°C for 30 min.

Instrument

Flow cytometric data were collected using NovoCyte (ACEA bioscience. Inc).

Software

NovoExpress 1.4.1.

Cell population abundance

We performed the flow cytometry for phenotypes of the memory T cells with the production of IFN-gamma or IL-4 by analysing the stimulated lymphocytes in the present study, and is easy to get enough CD8+ or CD4+ cell for further gating memory cells.

Gating strategy

Cells were gated from MHC-II- and CD45R-, CD4+ or CD8+ cells with CD44+ were defined as the memory cells reactive to RBD protein. From these gated cells the percentage of IFN-gamma and IL-4 positive cells were recorded.
ACE2 transfected-Cells with PE fluorescence intensity were counted for the inhibition of receptor binding assays.

☐ Tick this box to confirm that a figure exemplifying the gating strategy is provided in the Supplementary Information.

Magnetic resonance imaging

Experimental design

Design type

Indicate task or resting state; event-related or block design.

Design specifications

Specify the number of blocks, trials or experimental units per session and/or subject, and specify the length of each trial or block (if trials are blocked) and interval between trials.

Behavioral performance measures

State number and/or type of variables recorded (e.g. correct button press, response time) and what statistics were used to establish that the subjects were performing the task as expected (e.g. mean, range, and/or standard deviation across subjects).

Acquisition

Imaging type(s) — *Specify: functional, structural, diffusion, perfusion.*

Field strength — *Specify in Tesla*

Sequence & imaging parameters — *Specify the pulse sequence type (gradient echo, spin echo, etc.), imaging type (EPI, spiral, etc.), field of view, matrix size, slice thickness, orientation and TE/TR/flip angle.*

Area of acquisition — *State whether a whole brain scan was used OR define the area of acquisition, describing how the region was determined.*

Diffusion MRI ☐ Used ☐ Not used

Preprocessing

Preprocessing software — *Provide detail on software version and revision number and on specific parameters (model/functions, brain extraction, segmentation, smoothing kernel size, etc.).*

Normalization — *If data were normalized/standardized, describe the approach(es): specify linear or non-linear and define image types used for transformation OR indicate that data were not normalized and explain rationale for lack of normalization.*

Normalization template — *Describe the template used for normalization/transformation, specifying subject space or group standardized space (e.g. original Talairach, MNI305, ICBM152) OR indicate that the data were not normalized.*

Noise and artifact removal — *Describe your procedure(s) for artifact and structured noise removal, specifying motion parameters, tissue signals and physiological signals (heart rate, respiration).*

Volume censoring — *Define your software and/or method and criteria for volume censoring, and state the extent of such censoring.*

Statistical modeling & inference

Model type and settings — *Specify type (mass univariate, multivariate, RSA, predictive, etc.) and describe essential details of the model at the first and second levels (e.g. fixed, random or mixed effects; drift or auto-correlation).*

Effect(s) tested — *Define precise effect in terms of the task or stimulus conditions instead of psychological concepts and indicate whether ANOVA or factorial designs were used.*

Specify type of analysis: ☐ Whole brain ☐ ROI-based ☐ Both

Statistic type for inference (See Eklund et al. 2016) — *Specify voxel-wise or cluster-wise and report all relevant parameters for cluster-wise methods.*

Correction — *Describe the type of correction and how it is obtained for multiple comparisons (e.g. FWE, FDR, permutation or Monte Carlo).*

Models & analysis

n/a	Involved in the study
☐	☐ Functional and/or effective connectivity
☐	☐ Graph analysis
☐	☐ Multivariate modeling or predictive analysis

Functional and/or effective connectivity — *Report the measures of dependence used and the model details (e.g. Pearson correlation, partial correlation, mutual information).*

Graph analysis — *Report the dependent variable and connectivity measure, specifying weighted graph or binarized graph, subject- or group-level, and the global and/or node summaries used (e.g. clustering coefficient, efficiency, etc.).*

Multivariate modeling and predictive analysis — *Specify independent variables, features extraction and dimension reduction, model, training and evaluation metrics.*
